# Systematic revision and biogeography of the endemic *Lucanus kanoi* species complex (Coleoptera, Lucanidae) from Taiwan, with the description of a new subspecies

**DOI:** 10.3897/zookeys.1267.160494

**Published:** 2026-01-22

**Authors:** Shu-Ping Wu, Yu-Fang Tsai, Ting-Yang Chien, Yi-Ting Chung, Ching-Jung Lai, Tsung-Hsien Hou, Chung-Chi Hwang

**Affiliations:** 1 Department of Earth and Life Sciences, University of Taipei, Taipei, 100234, Taiwan National University of Kaohsiung Kaohsiung Taiwan; 2 Department of Physics, National Taiwan University, Taipei, 106319, Taiwan University of Taipei Taipei Taiwan; 3 Taitung Branch, Forestry and Nature Conservation Agency, Ministry of Agriculture, Taitung 950205, Taiwan National Taiwan University Taipei Taiwan; 4 Taipei Municipal Sanmin Junior High School, Taipei, 114059, Taiwan Ministry of Agriculture Taitung Taiwan; 5 Department of Life Sciences, National University of Kaohsiung, Kaohsiung, 811726, Taiwan Taipei Municipal Sanmin Junior High School Taipei Taiwan

**Keywords:** Biogeography, *Lucanus kanoi*, *Lucanus kanoi kavulunganus* subsp. nov., species complex, systematic revision

## Abstract

In this study, the taxonomic status of the *Lucanus kanoi* species complex is examined through a comprehensive systematic revision, and the phylogenetic relationships among its constituent taxa are investigated. Three taxa are analyzed: *L. kanoi*, *L. piceus*, and *L. ogakii*. Although closely related, the results support the recognition of each as a distinct species-level taxon. Specifically, *L. piceus***stat. nov**. is elevated to full species status, rather than being treated as a subspecies of *L. kanoi*. Furthermore, a new subspecies, *L. kanoi kavulunganus***subsp. nov**., is described based on consistent morphological, biogeographical, and molecular evidence.

## Introduction

Among the vast diversity of Coleoptera, stag beetles (family Lucanidae) represent a morphologically and behaviorally remarkable lineage. These beetles gained prominence in the field of natural history as early illustrations in Darwin (1871) exemplified his theory of sexual selection.

Taiwan hosts more than 50 species of stag beetles, exhibiting notable morphological diversity and ecological differentiation. These species inhabit a wide range of habitats, from tropical broadleaf forests at low elevations to temperate, old-growth forests in mid-elevation mountain zones (Kurosawa 1966; Imanishi 1990; Wang 1990, 1994; Chang 1993, 2006, 2024; Sakaino and Yu 1993; Mizunuma and Nagai 1994; Fujita 2010; Huang and Chen 2010; Huang 2016). Among them, the genus *Lucanus* is perhaps the most recognizable, with eleven species and subspecies currently reported from Taiwan (Scopoli 1763). However, the taxonomic status of several taxa within this genus remains controversial (Huang and Chen 2010; Chang 2024).

Early taxonomic descriptions were often based on a single or limited number of type specimens and lacked consideration of intraspecific morphological variation. Additionally, many diagnoses relied on vague or ambiguous terminology. For example, when Kurosawa (1966) described *L. k. piceus* as a subspecies of *L. kanoi*, only a single holotype and a single paratype were designated, and these two specimens originated from different localities. The morphological description in the original article was very brief and did not include any female specimens. Similarly, when the subspecies *L. ogakii chuyunshanus* was established, it was based on a single holotype without any paratypes, and the morphological description provided was likewise very limited (Imanishi 1990). As a result, the validity of several species names has been subject to ongoing debate, highlighting the need for a comprehensive and integrative systematic revision.

**Figure 1.**
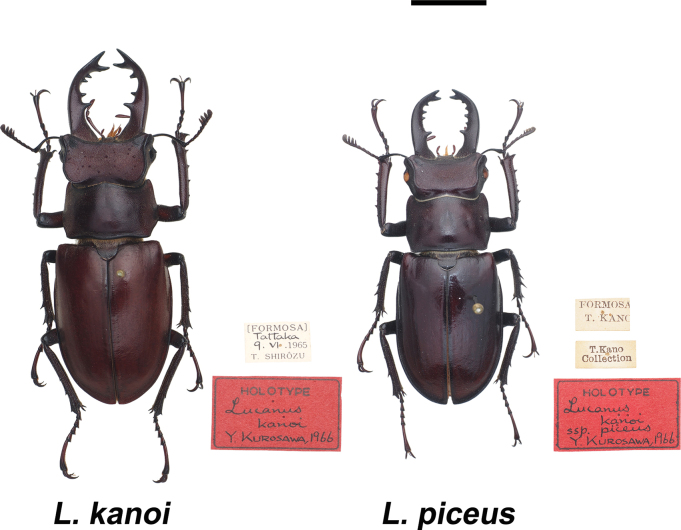
Type specimens of *Lucanus kanoi* and *L. k. piceus* deposited in NMNS, Tokyo. Photo by H. Akimoto and M. Hoso. Scale bar: 1 cm.

**Figure 2.**
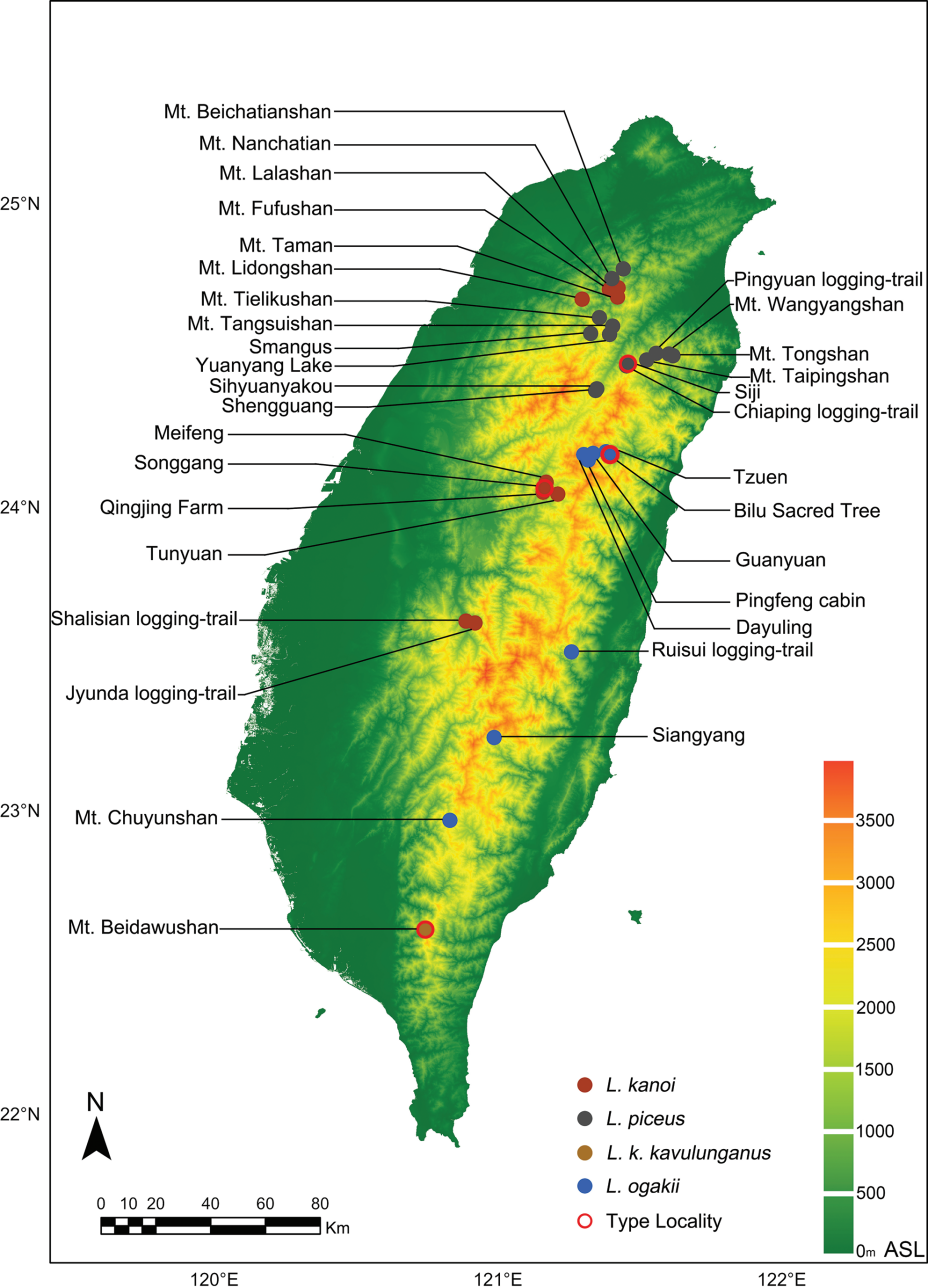
Type localities and sampling sites of *L. kanoi* species complex in this study. No specimens of *L. ogakii* from Mt. Chuyunshan were available for this study; the localities on the map are taken from Sakaino and Yu (1993).

Kurosawa (1966) described *Lucanus kanoi* based on 16 specimens collected from central Taiwan. In the same publication, he also described a subspecies, *L. kanoi piceus*, using two male specimens, one collected by T Kano from “Kuhsha” (today Siji, Fig. 2, Table 1) and another from Mt. Lalashan (collector unknown), both housed in the National Science Museum, Tokyo. Kuhsha was recorded as the type locality of *L. k. piceus*, represented solely by the holotype; the specimen from Mt. Lalashan was recorded as the paratype. The subspecies was distinguished primarily by its darker coloration (Kurosawa 1966) (Figs 1, 2, Tables 1, 2).

**Table 1.** Historical and current names of type localities for the *Lucanus kanoi* species complex.

Species	*L. kanoi*	*L. piceus* (formerly referred to as *L. k. piceus*)	*L. ogakii*
Type locality Historical name	Sungkang (Tattaka), Nantou Pref.	Kuhsha, I-Lan Pref. (holotype)	Piru, Hualian Pref.
		Mt. Lalashan, Taipei Pref. (Paratype)	
Current name	Songgang, Nantou County	Siji, Yilan County (holotype)	Bilu Sacred Tree, Hualien County
		Lalashan, Taoyuan City (paratype)	
GPS Coordinate	24°04.22'N, 121°10.03'E	24°28.82'N, 121°28.15'E	24°10.85'N, 121°24.20'E
		24°43.84'N, 121°26.05'E	
Altitude (meters, a.s.l.)	2030	1989	2180
		2031	

**Table 2.** Sampling localities of this study.

Species	Distribution		Altitude (meters, a.s.l.)	Acronyms / Remarks
	Locality	GPS Coordinates		
*Lucanus k. kanoi*	Songgang, Nantou County	24°04.22'N, 121°10.03'E	2030	SG / **Type locality**
*L. k. kanoi*	Mt. Lalashan, Taoyuan City	24°43.84'N, 121°26.05'E	2031	LLS
*L. k. kanoi*	Mt. Fufushan, Taoyuan City	24°43.50'N, 121°24.14'E	1870	FFS
*L. k. kanoi*	Mt. Tamanshan, Taoyuan City	24°42.00'N, 121°25.93'E	1800	TMS
*L. k. kanoi*	Mt. Lidongshan, Hsinchu County	24°41.58'N, 121°18.22'E	1914	LDS
*L. k. kanoi*	Mt. Shimatashan, Hsinchu County	24°37.53'N, 121°14.99'E	1942	SMTS
*L. k. kanoi*	Meifeng, Nantou County	24°05.41'N, 121°10.44'E	2100	MF
*L. k. kanoi*	Qingjing Farm, Nantou County	24°03.49'N, 121°09.75'E	1900	QJ
*L. k. kanoi*	Tunyuan, Nantou County	24°03.06'N, 121°12.92'E	2020	TY
*L. k. kanoi*	Jyunda logging-trail, Nantou County	23°37.56'N, 120°55.09'E	1990	JD
*L. k. kanoi*	Shalisian Logging-trail, Nantou County	23°31.74'N, 120°54.26'E	1910	SLS
*L. k. kavulunganus* subsp. nov.	Mt. Beidawushan, Pingtung County	22°36.93'N, 120°44.53'E	2140	BDW / **Type locality**
*L. piceus*	Siji, Yilan County	24°28.82'N, 121°28.15'E	1989	SJ / **Type locality**
*L. piceus*	Mt. Lepeishan, New Taipei City	24°48.67'N, 121°28.35'E	1569	LPS
*L. piceus*	Mt. Beichatianshan, New Taipei City	24°47.58'N, 121°27.16'E	1640	BCT
*L. piceus*	Mt. Nanchatian, Taoyuan City	24°45.65'N, 121°24.73'E	1907	NCT
*L. piceus*	Mt. Tielikushan, Taoyuan City	24°37.96'N, 121°21.99'E	1800	TLS
*L. piceus*	Mt. Tangsuishan, Taoyuan City	24°36.25'N, 121°24.84'E	2090	TSS
*L. piceus*	Smangus, Hsinchu County	24°34.80'N, 121°20.07'E	1576	SMG
*L. piceus*	Yuanyang Lake, Hsinchu County	24°34.62'N, 121°24.15'E	1600	YYL
*L. piceus*	Pingyuan logging-trail, Yilan County	24°30.84'N, 121°34.24'E	2000	PYLT
*L. piceus*	Mt. Wangyangshan, Yilan County	24°30.67'N, 121°36.98'E	2050	WYS
*L. piceus*	Mt. Tongshan, Yilan County	24°30.35'N, 121°37.86'E	1815	TS
*L. piceus*	Mt. Taipingshan, Yilan County	24°29.54'N, 121°32.20'E	1877	TPS
*L. piceus*	Chiaping logging-trail, Yilan County	24°28.36'N, 121°27.84'E	1940	CPLT
*L. piceus*	Sihyuanyakou, Yilan County	24°23.84'N, 121°21.39'E	1965	SYYK
*L. piceus*	Shengguang, Taichung City	24°23.52'N, 121°21.14'E	1960	SHG
*L. ogakii*	Bilu Sacred Tree, Hualien County	24°10.85'N, 121°24.20'E	2180	BL **Type locality**
*L. ogakii*	Tzuen, Hualien County	24°11.43'N, 121°23.34'E	1960	TE
*L. ogakii*	Guanyuan, Hualien County	24°11.10'N, 121°20.50'E	2330	GY
*L. ogakii*	Dayuling, Hualien County	24°10.85'N, 121°18.60'E	2564	DYL
*L. ogakii*	Pingfeng cabin, Hualien County	24°09.78'N, 121°19.51'E	2062	PFC
*L. ogakii*	Ruisui logging-trail, Hualien County	23°31.82'N, 121°15.81'E	2020	RS
*L. ogakii*	Siangyang, Taitung County	23°14.91'N, 120°59.21'E	2280	SY

Although the original description of *L. k. piceus* included only two male specimens and no females, subsequent literature has generally assumed that specimens from central Taiwan correspond to *L. kanoi*, while darker individuals from northern and northeastern Taiwan represent *L. k. piceus*.

In 2010, *Lucanus ogakii* Imanishi, 1990, a species closely related to *L. kanoi*, was reclassified as a subspecies of *L. kanoi* (*L. kanoi ogakii*) based on comparative morphology of the male external genitalia characteristics (Huang and Chen 2010). While this taxonomic decision was supported by genitalia morphology, it remains contentious and has not been universally accepted in later studies (Huang 2016; Chang 2024), especially in the absence of supporting molecular data.

Tsai and Yeh (2016) attempted to clarify the species-subspecies relationship between *Lucanus kanoi* and *L. k. piceus* using molecular data. However, their study did not accurately resolve the current geographical distributions of the two taxa. Although based on only a limited number of specimens from few localities, that study nevertheless inferred a wide geographical distribution for the two taxa in a speculative manner, which may lead to potential overlap in the geographical scope of sampling or to misidentification of specimens. Moreover, the study did not include type locality specimens as taxonomic reference. As a result, although their work discussed possible genetic boundaries between these taxa, it provided limited contributions toward resolving the taxonomic status and biogeographical delineation of *L. kanoi* and *L. k. piceus*.

Given these unresolved issues, a comprehensive re-examination of the *L. kanoi* species complex, encompassing its putative subspecies and closely related taxa is warranted. This study presents a systematic revision of the group, integrating morphological, biogeographical, and molecular phylogenetic evidence to clarify the relationships and taxonomic boundaries within the complex.

## Materials and methods

### Sampling

Samples were collected using light traps during May and June from 34 localities across Taiwan Island between 2020 and 2024. The sampling sites ranged from 1,569 to 2,564 meters above sea level, and included the type localities of *L. kanoi*, *L. k. piceus*, and *L. ogakii* (Fig. 2, Tables 1, 2, Suppl. material 1). All field-collected stag beetle specimens were legally obtained under research permits; the permit license numbers are listed in Suppl. material 2. In addition to field-collected specimens, material from museums were also examined. The acronyms of these museums are listed in Suppl. material 3.

In total, 985 specimens were examined in this study, including 407 *L. kanoi*, 261 *L. k. piceus*, and 298 *L. ogakii* (including two males and two females marked “*L. ogakii chuyunshanus*” from MSME, Suppl. material 4). The type specimens of *L. kanoi* and *L. k. piceus*, originally described by Kurosawa in 1966 and preserved at the National Science Museum (Tokyo), today known as the National Museum of Nature and Science, Tokyo (NMNS (JP)), were also examined in this study (Fig. 1, Suppl. materials 1, 3). Since all type specimens of *L. ogakii* formed parts of private collections and were not deposited in any public museum (Imanishi 1990), we were unable to examine the original types in the museum collection. Instead, we collected specimens from the type locality for analysis in this study (Fig. 2, Tables 1, 2).

A controversial subspecies of *L. ogakii*, named *L. ogakii chuyunshanus* (Sakaino & Yu, 1993), was described based on a single specimen collected from Mt. Chuyunshan in Kaohsiung, southern Taiwan (Fig. 2). According to the original publication, the holotype was deposited in the Muh Sheng Museum of Entomology (MSME) (Sakaino and Yu 1993). However, the holotype has since been lost and is no longer available, presumably due to damage sustained by MSME during the 1999 “921 Earthquake” in Taiwan. We examined four specimens exhibited at MSME, two males and two females labeled as *L. o. chuyunshanus*. However, none of these specimens has original collection labels, and there is no evidence confirming that they were collected from Mt. Chuyunshan (Suppl. material 4). Furthermore, no *L. ogakii* specimens have been collected from Mt. Chuyunshan in recent years, nor were any such specimens found in other museum or private collections examined during this study. Currently, it is generally believed that the *L. ogakii* population found in the Siangyang area may correspond to the subspecies *L. o. chuyunshanus*, largely due to the geographical proximity between Siangyang and Mt. Chuyunshan (Tsai and Yeh 2016; Chang 2024).

### Molecular phylogenetic analyses

To identify genetically delineated species, we employed taxonomic DNA barcoding using the mitochondrial cytochrome c oxidase subunit I (CO1) fragment as a molecular genetic marker (Moritz and Cicero 2004; DeSalle and Goldstein 2019). For each monophyletic clade recovered in the phylogenetic analyses, at least three specimens were selected for CO1 sequencing to ensure the stability of the tree topology. In cases where multiple specimens originated from geographically proximate localities and shared identical or highly similar CO1 sequences, one locality was selected to represent the group in downstream analyses to avoid redundancy. For example, specimens from LLS were found to belong to the same monophyletic group as those from TMS and FFS, and thus LLS was chosen as the representative. Similarly, JD was selected to represent the same clade as specimens from SLS. The acronyms of sampling localities are listed in Table 2.

Genomic DNA was extracted from thoracic muscle tissue of fresh specimens following the protocol of Truett et al. (2000). To assess phylogenetic relationships among the three subtypes, three gene regions were targeted: the mitochondrial CO1, mitochondrial 16S ribosomal RNA (16S rRNA), and the nuclear Wingless gene (Wnt). Partial fragments of CO1, 16S rRNA, and Wnt were amplified using the primer pairs LCO1491/HCO2198 (Folmer et al. 1994), 16Sar/16Sbr (Palumbi 1996a, b; Vences et al. 2005), and Wg550F/WgAbrZ (Wild and Maddison 2008), respectively. PCR amplifications were performed in 50 μl reaction volumes containing 5 μl of 10× reaction buffer (15 mM MgCl_2_), 4 μl of dNTPs (2.5 mM), 0.05 U of Super-Therm Taq polymerase, 0.5 μl of each primer (10 pmol/μl), 1 μl of template DNA, and nuclease-free water. Thermal cycling conditions included an initial denaturation at 95 °C for 5 min; followed by 35–40 cycles of denaturation at 95 °C for 1 min, annealing at 48–50 °C for 1 min, and extension at 72 °C for 1 min; with a final extension at 72 °C for 7 min. PCR products were visualized in 2% agarose gels, purified using a Geneaid PCR Extraction Kit (Geneaid DF 100), and sequenced on an ABI 3730 automated sequencer. The GenBank accession numbers of the DNA sequences generated in this study are provided in Suppl. material 5. In addition, molecular sequences of related species already deposited in GenBank were also incorporated and integrated into the comparative analyses.

Chromatographs and sequences were examined, compiled, edited, and converted between formats in AliView 1.26 (Larsson 2014). Sequences were aligned using MAFFT (Katoh and Standley 2013). Both the 5’ and 3'Ends were trimmed to avoid missing sites. In total, 1598 bp of concatenated sequences were used for phylogenetic analyses, including 507 bp of 16S, 644 bp of CO1, and 447 bp of Wnt. The uncorrected proportional distances (p-distance) between sequences and taxa were calculated using MEGA 10 (Kumar et al. 2018).

The best-fit nucleotide substitution model for each gene partition was selected using ModelFinder (Kalyaanamoorthy et al. 2017) implemented in IQ-TREE v. 2.1.3 (Nguyen et al. 2015), based on the Bayesian Information Criterion (BIC). The selected models were HKY+Γ+I for 16S and COI, and GTR+Γ+I for Wnt. These models were subsequently applied in partitioned Bayesian inference (BI) using the Markov chain Monte Carlo (MCMC) method implemented in MrBayes v. 3.2.6 (Ronquist et al. 2012), and in maximum likelihood (ML) analysis performed in IQ-TREE v. 2.1.3 (Nguyen et al. 2015). For BI, four independent runs with four chains each were executed for 2 million generations, with trees sampled every 500 generations. Convergence was assessed in Tracer v. 1.7.1 (Rambaut et al. 2018) and considered adequate when the effective sample size (ESS) for all parameters exceeded 200. The first 25% of samples were discarded as burn-in, and a 50% majority-rule consensus tree was constructed, with posterior probabilities (PP) calculated for each branch. Branch support in ML analyses was assessed with 5000 non-parametric fast bootstrap replicates. Preliminary phylogenetic analyses were conducted using additional sequences retrieved from GenBank to determine adequate sampling for phylogenetic analyses of the group (Tsai and Yeh 2016).

### Divergence time estimation

Divergence time was estimated by using the Bayesian Markov chain Monte Carlo algorithm complemented in BEAST 2 (Bouckaert et al. 2014). The best-fit substitution model for 16S and CO1, HKY+Γ+I, was applied for the following analysis. The strict molecular clock was employed for the BEAST analysis. Substitutions rates for beetles (Papadopoulou et al. 2010) were used, that is, 0.0054 per site per million years for 16S and 0.0177 per site per million years for CO1. The BEAST analysis was run for 20 million generations, with a sampling frequency set to 1,000. The convergence of runs was judged by using Tracer v. 1.7.1, when the ESS for all parameters were more than 200. A maximum clade credibility tree with median node heights was calculated with the TreeAnnotator utility compiled in BEAST. One-fourth of the sample trees were discarded by burn-in.

### Morphological characteristics and analysis

**Figure 3.**
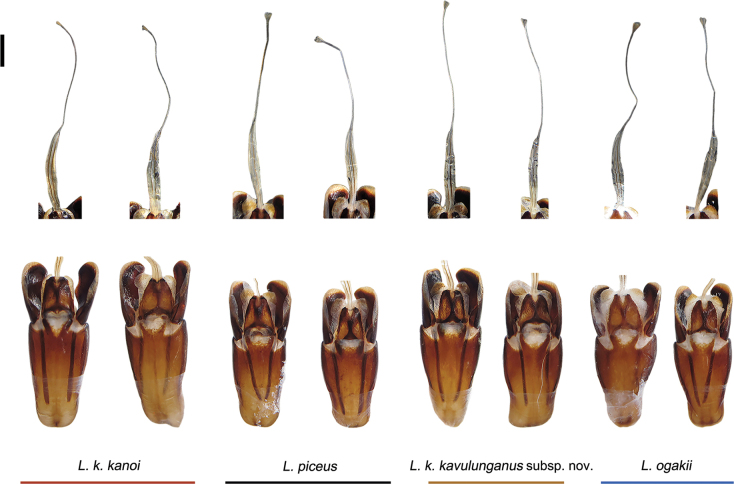
Comparative anatomy of the male genitalia of *L. k. kanoi* and related species. The flagellum is shown above, and the basal piece below. Note that the basal piece of *L. k. kanoi* is slightly larger than that of *L. piceus*, with no other obvious morphological differences. Photographs by H. Huang. Scale bar: 1 mm.

Due to the brevity of the original descriptions and the limited number of type specimens, morphological information available in the initial taxonomic treatments is scarce. In the present study, descriptions of external and genital morphology are partially derived from earlier works (Kurosawa 1966; Imanishi 1990), but primarily follow the detailed accounts provided by Huang and Chen (2010), which currently offer the most comprehensive morphological treatment of these taxa. Comparative examinations of the morphological characteristics of the male genitalia were performed with reference to anatomical data provided by Mr. H. Huang (pers. comm. 05 Dec. 2024; Fig. 3).

A total of 841 male individuals from three taxa were analyzed: *L. ogakii* (*n* = 254), *L. kanoi* (*n* = 359), and *L. piceus* (*n* = 228). Due to the high degree of external morphological similarity among females of the three taxa, it is difficult to distinguish interspecific differences based on morphological traits (Huang and Chen 2010; Table 3). Therefore, morphological analyses in this study were conducted using male specimens only.

**Table 3.** Key traits of the *Lucanus kanoi* species complex reported in the literature.

Species Characteristics	*Lucanus kanoi kanoi*	*Lucanus piceus*	*Lucanus kanoi kavulunganus*. subsp. nov.	*Lucanus ogakii*	Literature
**Male**					1. Kurosawa 1966
					2. Imanishi 1990
					3. Sakaino and Yu 1993
					4. Chang 2006
					5. Fujita 2010
					6. Huang and Chen 2010
					7. Huang 2016
					8. Chang 2024
					9. This study
Clypeolabrum	Lateral ridges and frontal ridge not markedly protruding^1^. Longer and narrower, with labrum not clearly separated from clypeus at lateral sides^6.^ Absence of lateral angles of the clypeus^6^	Shorter and wider, with labrum clearly separated from clypeus at lateral sides by the clear marked lateral angles of clypeus^6.^ Indistinct frontal ridge, and the flat and non-cupped vertex behind the frontal ridge of the head^6^	Similar to *L. kanoi* but slightly smaller^9^	Clypeus is broad, not projecting acutely at apex^2^.	
Coloration	Chocolate-brown^1^	Body blackish, piceous^1^. Various colors, included blackish & piceous or reddish brown^6^	Blackish to reddish-brown^9^	Body entirely reddish-brown^3^. Reddish brown dorsal surface^6^	
Elytron tint	Reddish-brown or blackish^6^	Elytra more nitidous, more sparsely covered with finer smaller punctures^1^	Blackish or reddish-brown, nitidous^9^	Elytra are broad and rounded^2.^ Elytra strongly tinged with red^3.^ Black or brownish red elytra^6^	
Elytron texture	Glabrous, inconspicuous hairs^1^. The more hairy and less shining dorsal surface of body^6^	More shining, hair of elytra finer and more inconspicuous^1^.	Glossy^9^	Glossy^9^	
Mandible and Tooth	Shorter, more robust, more strongly arcuate. Apical teeth sharply and similarly furcate, sparse denticles with well-marked apical fork^1^. Basal tooth not closely associated with other teeth^6^		The basal tooth of the mandible is situated approximately at the basal one-third to one-fourth of its length^9^	Only one internal tooth in mandibles^2^	
	General description: 1) mandible nearly straight or slightly incurved from base to middle and markedly incurved at middle or a little beyond middle; 2) basal tooth of the mandibles well separated from the neighboring tooth, not closely associated with other teeth; 3) basal tooth of the mandible usually closer to base; 4) clypeolabrum protruding well or at least with lateral angles of the clypeus clearly beyond the inner margin of the mandibles; 5) dorsal surface of the whole body with a markedly shorter and sparser pubescence^6^				
Leg	Most individuals have dark, blackish to brown legs similar to the coloration of ventral side of the body, 21.6% individuals have a yellowish to dark orange plaque at the underside of femur^9^	Most individuals have dark, blackish to brown legs similar to the coloration of ventral side of the body, approximately 13.2% individuals have a yellowish to dark orange plaque at the underside of femur^9^	Most individuals have dark, blackish to brown legs similar to the coloration of ventral side of the body, 31.58% individuals have a yellowish to dark orange plaque at the underside of femur^9^	Femora and tibiae are black^2^. Femora distinctly ornamented with yellowish brown stripes on the under surface^3^. Femora black or with yellowish brown strips^6^. Most individuals have dark, blackish to brown legs similar to the coloration of ventral side of the body, 1.22% individuals have a yellowish to dark orange plaque at the underside of femur^9^	
Genitalia	1) Cephalic process of the paramere in dorsal view **stout**; 2) apical duct / basal belt in flagellum 1.3-1.4^6^	1) Cephalic process of the paramere in dorsal view **stout**; 2) apical duct / basal belt in flagellum 1.4^6^	1) Cephalic process of the paramere in dorsal view **stout**; 2) apical duct / basal belt in flagellum 1.3-1.4^6^	1) Cephalic process of the paramere in dorsal view **slender**; 2) apical duct / basal belt in flagellum 1.8^6^	
	1) ventral plate of the basal piece clearly marked and rather long, with middle part shallowly and well beyond caudal margin of the basal piece; 2) ventral plate of the ninth abdominal segment markedly constricted before the caudal expansion; 3) flagellum length similar, without significant difference; 4) apex of paramere in lateral view not hollowed^6^				
**Female**					
General description	1) inner tooth of the left mandible single-pointed; 2) mesofemora and metafemora uniform dark; 3) posterior margin of the canthus more or less marked; 4) punctures on the head larger and coarser^6^				
Head	Anterior angles of the head usually indistinct^6^	Anterior angles of the head usually sharp or distinct^6^	Anterior angles of the head usually distinct^9^	Anterior angles of the head usually sharp or distinct^6^	
Mandible	With a distinct inferior tooth and a clear gap behind apex of mandible, the inner tooth without broad inner edge^1^				
Canthus	Without a distinct anterior angle^1^				
Pronotum	Anterior sides not strongly swollen, anterior angles sharp, rather broadly round, not angulate at the posterior third of the widest part^1^. Posterior angles not strongly angulate, rather rounded^1.^ Pronotum not wider at anterior 1/3 and evenly rounded here^6^	Pronotum not wider at anterior 1/3 and evenly rounded here^6^	At the anterior third, the pronotum does not expand laterally and maintains an evenly curved contour ^9.^ The posterior angles of the pronotum are not sharply angulate, but rather broadly rounded^9^. The pronotum is not noticeably widened at the anterior third and is evenly rounded in this region^9^	Pronotum often wider at anterior 1/3 and somewhat angled here^6^	
Metasternum	Metasternum with a shorter pubescence^6^	Metasternum with a shorter pubescence^6^	Metasternum with a shorter pubescence^9^	Metasternum sometimes with a longer pubescence^6^	
Leg	Entirely black^1^	Black^4,6,7,8,9^	Black^9^	Black^4,6,7,8,9^	
Genitalia	General description. *L. kanoi*, *L. piceus* and *L. ogakii* have no obvious difference in female genitalia^6^. 1) spermathecal duct nearly 2.5 times as long as spermatheca; 2) spermathecal duct 1.5 times as long as hemisternite; 3) spermatheca nearly 0.5-0.7 times as long as hemisternite; 4) spermatheca sclerotized; 5) spermathecal duct weakly sclerotized; 6) spermathecal gland markedly wider than spermathecal; 7) spermathecal gland nearly as long as spermatheca; 8) central conjunction of the ninth tergites weakly or markedly protruding posteriorly; 9) last abdominal tergite with lateral angles indistinct; 10) last abdominal ventrite excavated in middle^6^				
Body length max.-min. (mm)	M: 30-57 F: 24.58-43.97	M: 28.15-57 F: 28.27-43.94	M: 28.07-46.79^9^ F: 39.09-37.17^9^	M: 24.53-45.72 F: 23.93-38.99	Literature records and this study
Body length range (mm)	M: 30.30-52.86 F: 24.58-43.97	M: 28.15-51.05 F: 29.00-39.95	M: 28.07-46.79 F: 39.09-37.17	M: 25.04-42.53 F: 23.93-38.99	This study
Body length average (mm)	M: 39.92 ± 4.77 (n = 359) F: 32.39 ± 3.93 (n = 48)	M: 38.17 ± 4.48 (n = 228)F: 33.82 ± 3.27 (n = 33)	M: 36.22 ± 3.69 (n = 38) F: 34.53 ± 2.44 (n = 7)	M: 33.39 ± 3.34 (n = 254) F: 30.82 ± 3.48 (n = 41)	

**Figure 4.**
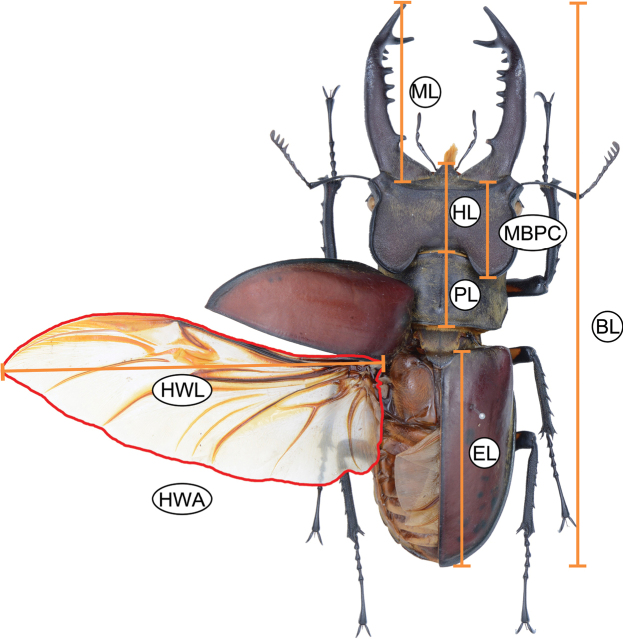
External morphological characters analyzed in this study. *Lucanus taiwanus* is used as a model species from the same genus, as its clypeal features are more distinct and more readily captured in photographs. Although not part of the focal taxa examined in this study, it provides a clear reference for morphological illustration. Abbreviations: BL: body length, EL: elytron length, PL: pronotum length, HL: head length, MBPC: Distance from mandible base to posterior margin of the clypeus, ML: mandible length, HWL: hind wing length, HWA: hind wing area.

We measured eight external morphological characters for each individual: body length (**BL**), elytron length (**EL**), pronotum length (**PL**), head length (**HL**), distance from mandible base to posterior clypeus margin (**MBPC**), mandible length (**ML**), hind wing length (**HWL**), and hind wing area (**HWA**) (Fig. 4). Recognizing the substantial intraspecific variation, particularly in male mandible morphology, that can obscure species boundaries when relying solely on absolute metrics, we also derived 28 ratio-based variables from all pairwise combinations of the eight raw measurements. The ratios of these 28 morphological characters are as follows: BL/EL, BL/PL, BL/HL, BL/MBPC, BL/ML, BL/HWL, BL/HWA, EL/ PL, EL/ HL, EL/ MBPC, EL/ ML, EL/HWL, EL/HWA, PL/ HL, PL/ MBPC, PL/ ML, PL/ HWL, PL/HWA, HL/MBPC, HL/ML, HL/ HWL, HL/HWA, MBPC/ ML, MBPC/ HWL, MBPC/HWA, ML/ HWL, ML/HWA, HWL/ HWA. This yielded a total of 36 candidate morphological traits. To reduce redundancy and multicollinearity among variables, we calculated the pairwise Pearson correlation coefficients for all 32 traits and retained 12 variables with correlation coefficients below 0.8. These included one raw and eleven ratio-based features: BL, BL/PL, BL/HL, BL/HWL, EL/PL, EL/HWL, PL/HWL, HL/MBPC, HL/ML, MBPC/ML, MBPC/HWA, and ML/HWA. Because many of the 36 traits are derived ratios of the same underlying measurements, they are algebraically dependent and thus not statistically independent variables. Applying PCA directly to all 36 traits would therefore give undue weight to redundant dimensions, inflate noise, and potentially obscure biologically meaningful patterns. By restricting the analysis to 12 carefully selected features, we minimized mathematical collinearity and ensured that the clustering reflected genuine morphological structure rather than artifacts of ratio construction.

We first conducted PCA on the 12 selected morphological variables to decorrelate traits and project specimens into an orthogonal morphometric space. K-means clustering was then performed on the resulting PCA scores. The optimal number of clusters was determined using the gap statistic. Final cluster assignments were based on k-means applied to the PCA-transformed data, and clusters were visualized using the first two principal components.

### Distribution and biogeography

ArcGIS software (ESRI 2011) was employed to generate landscape and phenological maps of Taiwan, with each collection locality plotted to illustrate the biogeographical distribution patterns of the examined taxa. Meteorological data, including isothermal diagrams used in the construction of the phenological map, were obtained from the open-access platforms CODiS (Climate Observation Data Inquire Service) and TCCIP (Taiwan Climate Change Projection Information and Adaptation Knowledge Platform). Monthly isotherm maps were derived from mean temperature records collected between 2010 and 2020, with data processing and compilation conducted by JY Lee (Lab. of Ecology and Evolution, Dep. of Earth and Life Sciences, UT).

## Results

### Molecular phylogeny, taxonomic implications, and divergence time

**Figure 5.**
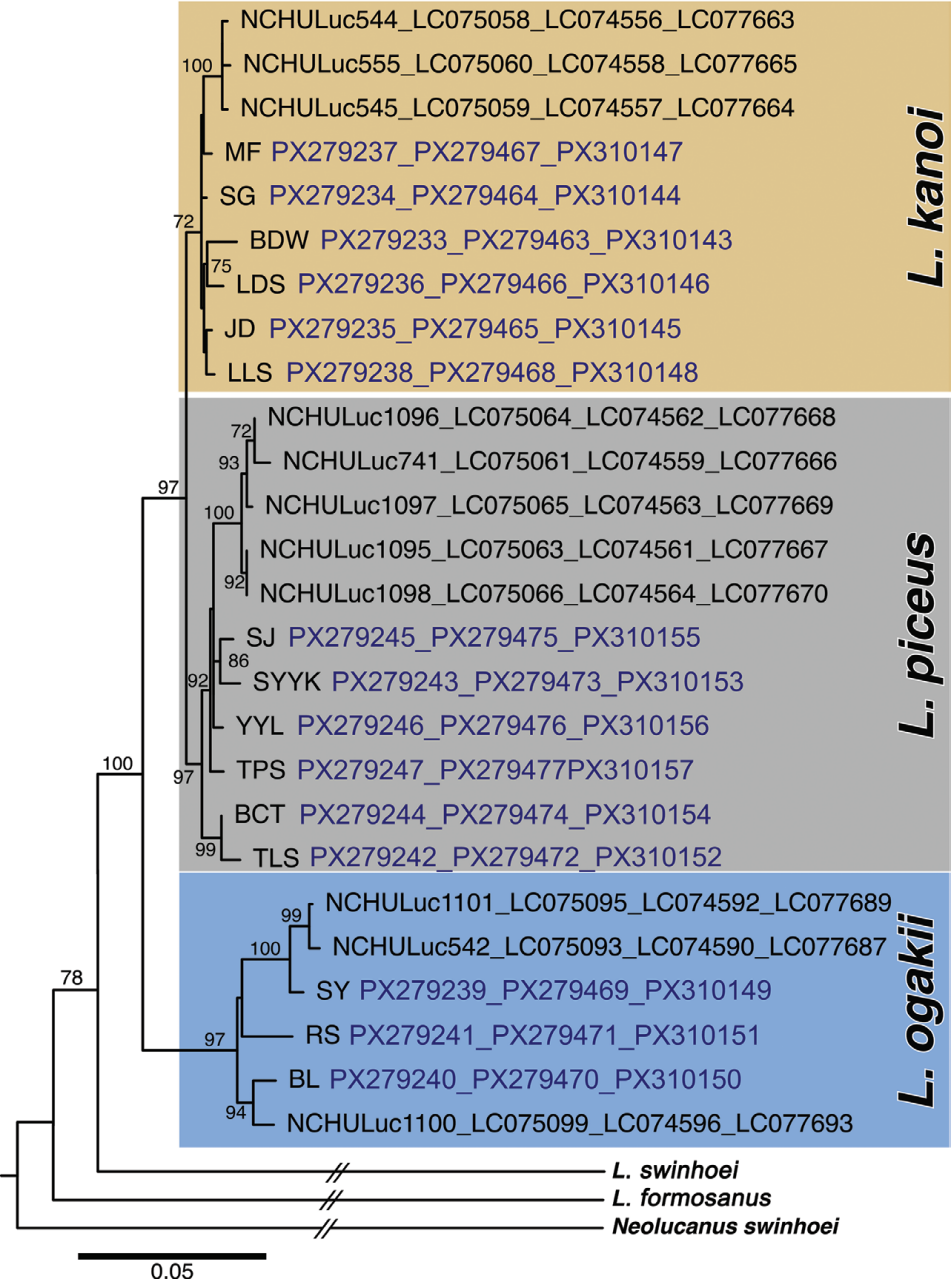
Inferred phylogenetic relationships of the *Lucanus kanoi* species complex. The phylogenetic tree was reconstructed using the ML method based on a 1,598 bp concatenated dataset, comprising 507 bp of 16S rRNA, 644 bp of COI, and 447 bp of the nuclear Wnt *gene*. Sequences retrieved from GenBank are shown with voucher numbers and corresponding accession numbers (in black). Numbers above branches indicate fast-bootstrap support values above 70%. The substitution models applied were HKY+Γ+I for 16S and GTR+Γ+I for COI and *Wnt.* Outgroups include *L. swinhoei*, *L. formosanus*, and *Neolucanus swinhoei*. Abbreviations: L.: *Lucanus*; N.: *Neolucanus*; SY: Siangyang; RS: Ruisui; BL: Bilu Sacred Tree; BCT: Beichatianshan; TLS: Tielikushan; TPS: Taipingshan; YYL: Yuanyang Lake; SJ: Siji; SYYK: Sihyuanyakou; LLS: Lalashan; MF: Meifeng; LDS: Lidongshan; SG: Songgang; JD: Jyunda; BDW: Beidawushan.

Molecular phylogenetic analyses revealed that *Lucanus kanoi* and its traditionally recognized subspecies *L. k. piceus* form two well-supported, reciprocally monophyletic clades. In contrast, specimens identified as *L. ogakii* clustered outside this lineage and formed the immediate outgroup (Fig. 5, Suppl. material 6). These results indicate that *L. ogakii* should not be treated as a subspecies or sub-lineage of *L. kanoi* clade, contrary to the conclusion of Huang and Chen (2010), who proposed that *L. ogakii* represents a southeastern subspecies of *L. kanoi*. Within the *L. kanoi*–*L. k. piceus* lineage, two distinct short-branched genetic clades were recovered: one of the clades includes specimens from Songgang (SG), the type locality of *L. kanoi*; the other clade contains specimens from Siji (SJ), the holotype locality of *L. k. piceus*. Therefore, given that each forms a distinct monophyletic clade, *L. kanoi* and *L. k. piceus* should be regarded as separate taxa. In the following sections, when describing *L. k. piceus*, we will refer to this taxon as *L. piceus*, which is raised to species level in this work.

Notably, the only paratype of *L. piceus* was collected from Mt. Lalashan. However, our analyses demonstrate that specimens from Mt. Lalashan (LLS) and the adjacent Mt. Tamanshan (TMS) group within the *L. kanoi* clade (Figs 2, 5, Tables 1, 2, Suppl. material 6). This indicates that the Lalashan paratype was misidentified and should be reassigned to *L. kanoi*. Despite this, the holotype of *L. piceus*, collected from Kuhsha (modern-day Siji), belongs to a distinct monophyletic lineage, thereby preserving the taxonomic validity of *L. piceus*. The Lalashan population, meanwhile, defines the northernmost known extent of the *L. kanoi* distribution (Figs 2, 5, Suppl. material 6).

A further noteworthy finding concerns a moderately sized, reddish to blackish population from Mt. Beidawushan in southern Taiwan. This population had previously been considered part of *L. ogakii* due to its geographic proximity to the Siangyang population in Taitung County (Fig. 2, Table 2). However, molecular data place the Beidawushan specimens within the *L. kanoi* clade. Given their geographic isolation and distinct genetic lineage, we propose recognizing the Beidawushan population as a new subspecies of *L. kanoi*, distinct from central and northern populations.

The estimated divergence times were approximately 1.25 million years ago (Mya; 95% HPD = 0.82–1.84 Mya) between *L. kanoi* and *L. piceus*, and 3.65 Mya (95% HPD = 2.55–5.05 Mya) between *L. ogakii* and the *L. kanoi*–*L. piceus* lineages. Based on the estimated divergence times, these three taxa should be regarded as distinct species rather than as species and subspecies within the same lineage (Suppl. material 7).

### Systematic account

**Family Lucanidae Latreille, 1804**

**Genus *Lucanus* Scopoli, 1763**

#### Lucanus kanoi kanoi

Taxon classificationAnimaliaColeopteraLucanidae

Kurosawa, 1966

4877CE29-7123-599A-A6AC-F92A0D04175B

Figs 1, 6

Lucanus kanoi Kurosawa, 1966: 339–344, pl. 1, figs 1–3. Wang 1990: 22–24; Chang 1993: 50, pl. 22, I3–1–I3–6, I3–8– 3–9; Wang 1994: 32–35; Chang 2006: 46–47; Mizunuma and Nagai 1994: 214, pl. 12, figs 100–1–5; Fujita 2010: 93, pl. 35, figs 234–1–7; Huang 2016: 112–115; Chang 2024: 44–45.
Lucanus kanoi kanoi Kurosawa, 1966: 339–344, pl. 1, figs 1–3. Huang and Chen 2010: 125–127, pl. 3, figs 11–16, pl. 4, fig. 60, pl. 15, figs 1, 2, pl. 29, fig. 1, pl. 40, figs 2, 3, pl. 51, fig. 17, pl. 55, fig. 8.

##### Type material examined.

***Holotype***. 1 male specimen, 52.22 mm, collected from Sungkang (today Songgang, Fig. 2, Table 1) by T. Shirôzu on Jun. 9, 1965. Deposited in NMNS, Tokyo (JP) (Fig. 1). 1 female ***allotype*** specimen; 6 males and 1 female ***paratype*** specimens, all type specimens were collected from the type locality (Kurosawa 1966).

**Figure 6.**
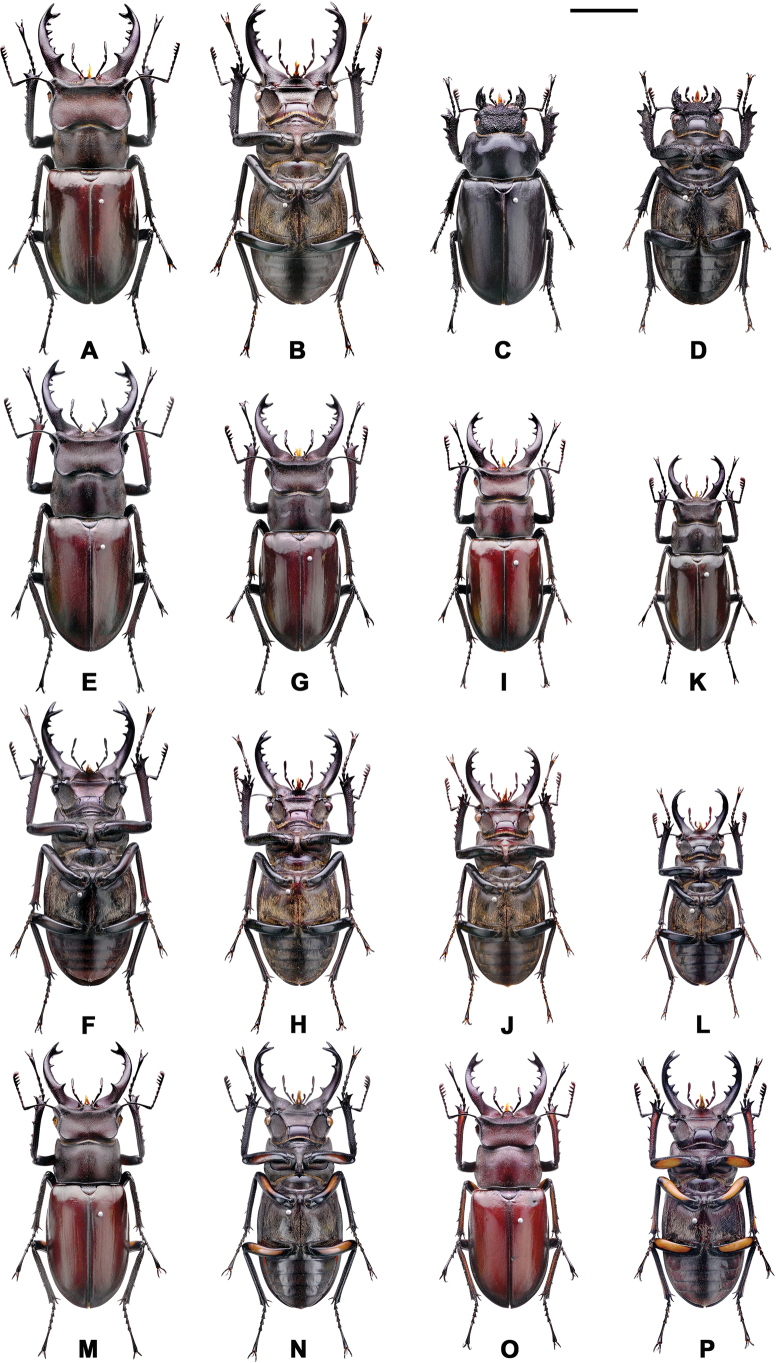
*Lucanus k. kanoi*, variation in gender, size, mandible, and color. **A, B, E, F**. Maximum form of male; **C, D**. Female; **G–J**. Medium form; **K, L**. Minimum form; **M–P**. With the femur orange plaque. Scale bar: 1 cm.

##### Additional material.

A total of 359 males (39.92 ± 4.77 mm, min-max 30–57 mm) and 48 females (32.39 ± 3.93 mm, min-max 24.58–43.97 mm) specimens collected from 10 localities were examined in this study including 35 individuals (31 male and 4 female) from the type locality (Fig. 2, Table 1). Variation of body size and color are presented in Fig. 6 and Table 3.

##### Diagnosis.

A moderately sized species of *Lucanus*, with the elytra brown to reddish or blackish in coloration. Sexual dimorphism is pronounced. Males measure 30.0–57.0 mm in body length, while females range from 24.58–43.97 mm. Males possess relatively short, robust mandibles with sharp apical teeth. The ventral surface is blackish with sparse fine hairs; legs are typically blackish, although a few individuals exhibit a yellowish to dark orange patch on the underside of the femora. Females have short mandibles, dark-brown to blackish elytra, and blackish femora. The species is primarily distributed in central Taiwan, with one population extending northward to Mt. Lalashan and its surrounding areas, including Mt. Tamanshan (Fig. 2, Table 2).

##### Description.

Male. 30.0–57.0 mm; female. 24.58–43.97 mm (Kurosawa 1966; Chang 2006; Huang and Chen 2010; Chang 2024; this study; Table 3). The coloration of the body, external morphology, and genital anatomy are illustrated in Figs 3, 6, and Table 3. Approximately 20.9% of males (75 of 359 individuals, including the holotype) exhibit a yellowish to orange plaque on the underside of the femora. However, this trait is variable and not sufficiently stable to serve as a reliable taxonomic character. Therefore, the presence of this plaque is regarded as an intraspecific variation within the species and its closely related taxa (Fig. 6, Table 3).

##### Distribution.

The species is mainly distributed in central Taiwan, with one population extending northward to Mt. Lalashan and its surrounding areas, including Mt. Tamanshan (Fig. 2, Table 2). Another recently discovered small population restricted to the high-elevation area of Mt. Beidawushan in southern Taiwan is described below as a new subspecies of *L. kanoi*.

##### Ecology.

This species inhabits primary broad-leaved forests at mid- to high elevations between 1800 and 2100 m (Fig. 2, Table 2). Adults are primarily active during June. By comparing the monthly average temperature isotherm in June with the altitudinal range of the species, it is inferred that this stag beetle tends to inhabit cooler environments (Suppl. material 8).

##### Conservation status.

Not evaluated (NE).

##### Remarks.

The systematic status of *L. kanoi* is consistent as belonging to a valid species. Previous literature identified the northern population of *L. kanoi* from Mt. Lalashan as belonging to the subspecies *L. kanoi piceus* (revised as *L. piceus* in this study). Based on molecular phylogenetic evidence, this long-standing misidentification is corrected in this work. Populations from Mt. Lalashan and the nearby Mt. Tamanshan should be assigned to *L. k. kanoi* (sensu stricto) rather than to *L. piceus* (Figs 2, 5, Suppl. material 6).

#### Lucanus piceus

Taxon classificationAnimaliaColeopteraLucanidae

Kurosawa, 1966 stat. nov.

4DB75A6A-4848-5D6A-8A73-755B9370C2EF

Figs 1, 7

Lucanus kanoi piceus Kurosawa, 1966: 342, pl. 1, fig. 4. Chang 1993: 50, pl. 22, I3–7; Mizunuma and Nagai 1994: 214, pl. 12, figs 100–6–7; Chang 2006: 46–47; Fujita 2010: 93, pl. 35, fig. 234–8–12; Huang and Chen 2010: 127–129, pl. 3, figs 17–21, pl. 4, fig. 61, pl. 15, fig. 4, pl. 29, fig. 2, pl. 40, figs 6–15, pl. 51, fig. 18, pl. 55, fig. 9; Huang 2016: 116–119; Chang 2024: 45.

##### Type material examined.

***Holotype***. 1 male specimen, 42.88 mm, collected from type locality Kuhsha (today Siji, Fig. 2, Table 1) by T. Kano 9 Jul. 1926. Deposited in NMNS (JP) (Fig. 1).

##### Additional material.

**Figure 7.**
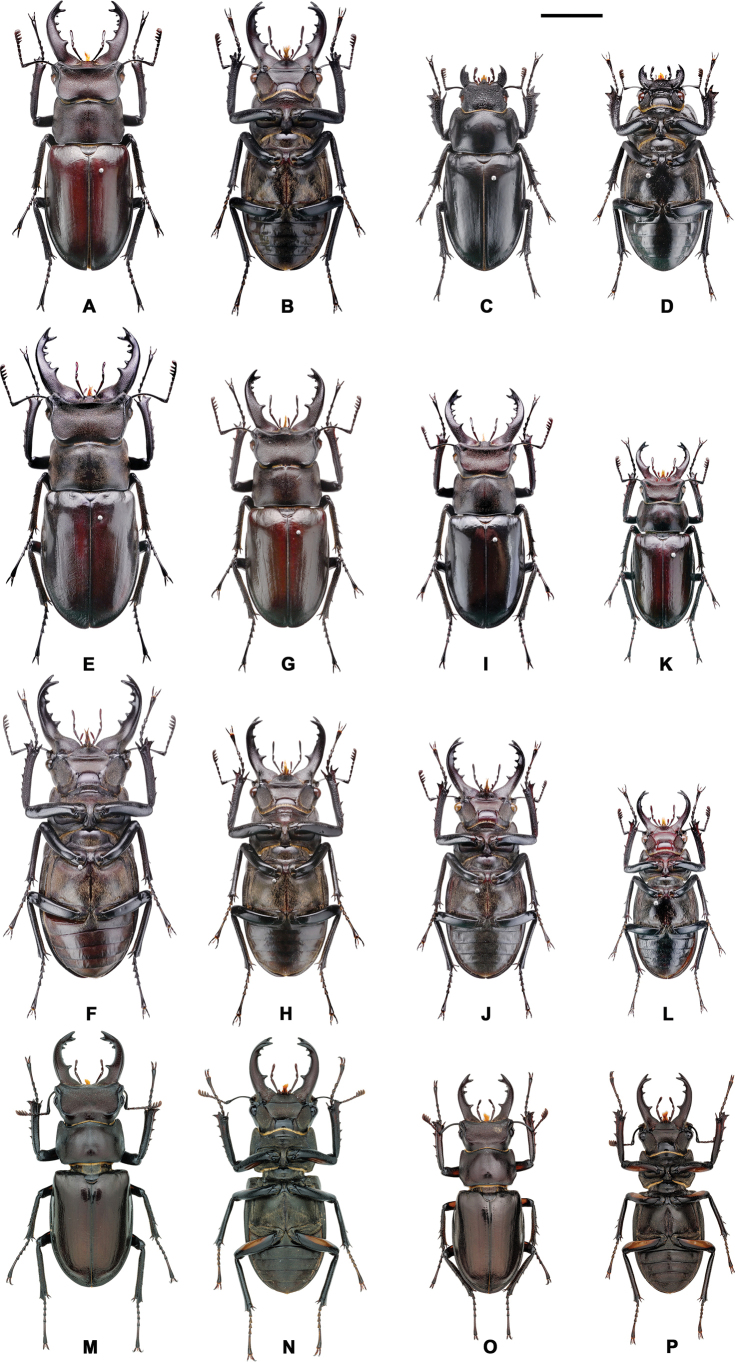
*Lucanus piceus*, variation in gender, size, mandible and color. **A, B, E, F**. Maximum form of male; **C, D**. Female; **G–J**. Medium form; **K, L**. Minimum form; **M–P**. With the orange plaque on the femur. Scale bar: 1 cm.

A total of 228 males (38.17 ± 4.48 mm, min-max 28.15–51.05 mm) and 33 females (33.82 ± 3.27 mm, min-max 29.00–39.95 mm) specimens collected from 14 localities were examined in this study including 103 individuals (90 males and 13 females) from type locality (Fig. 2, Suppl. material 1). Variation of body size and color are presented in Fig. 7 and Table 3.

##### Diagnosis.

A moderately sized *Lucanus*, with external morphology similar to *L. kanoi* but usually smaller in size. The elytra are reddish to blackish in color, glossy. Sexual dimorphism is pronounced, with males ranging from 28.15 to 57.0 mm in body length and females from 28.27 to 43.94 mm. The ventral surface is blackish with sparse fine hairs. Most individuals have blackish legs, while a few exhibit a yellowish to dark orange plaque on the underside of the femora. Females have short mandibles, dark-brown to blackish elytra, and blackish femora. This species is distributed in northern and northeastern Taiwan.

##### Description.

Males range from 28.15 to 57.00 mm, and females from 28.27 to 43.94 mm (Kurosawa 1966; Chang 2006; Huang and Chen 2010; Chang 2024; Table 3). Previous literature suggested that the most distinguishing characteristic of this species was its dark body coloration (Kurosawa 1966); however, this study confirms that the body color of this species varies significantly and cannot be used as a stable taxonomic characteristic. The variation in body color, external morphology, and genitalia anatomy is shown in Figs 3, 7, and Table 3. Approximately 13.2% of males (30/228, including the holotype) exhibit a yellowish to orange plaque on the femur, but this characteristic is not a consistent morphological feature and should not be used for taxonomic classification. Therefore, this plaque is considered a trait variation within the species and its related taxa.

##### Distribution.

This species is distributed in northern and northeastern Taiwan, where it inhabits primary broad-leaved forests at mid-to-high altitudes between 1569 and 2090 m (Fig. 2, Table 2).

##### Ecology.

Adults are primarily active in June. By comparing the monthly average temperature isotherms for June with the species’ altitudinal range, it is inferred that this stag beetle tends to inhabit cooler environments (Suppl. material 8).

##### Conservation status.

Not evaluated (NE).

##### Remarks.

The systematic status of *Lucanus kanoi piceus* Kurosawa, 1966 should be elevated from subspecies to species level, and is herein recognized as *Lucanus piceus* Kurosawa, 1966.

#### Lucanus kanoi kavulunganus subsp. nov.

Taxon classificationAnimaliaColeopteraLucanidae

B85B1799-021B-56BF-B08E-525FF4EBA7F1

https://zoobank.org/E2B73C17-1038-4F5E-9571-35EEB8CB6291

##### Type material.

**Figure 8.**
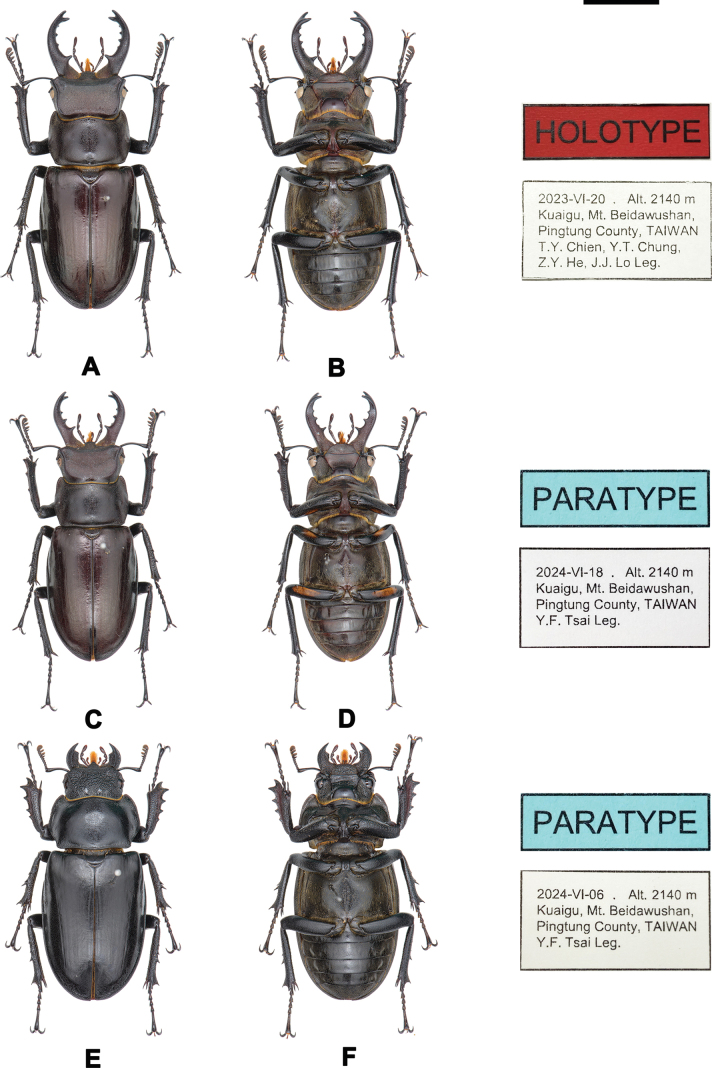
Type specimen of *Lucanus kanoi kavulunganus* subsp. nov. **A, B**. Holotype; **C, D**. Paratype 1 (male with orange plaque of femur); **E, F**. Paratype 2 (female). All type specimens presented here were from the type locality and deposited in Taiwan Agricultural Research Institute (TARI). Scale bar: 1 cm.

***Holotype***. An adult male (Fig. 8A, B), 41.44 mm, collected on Kuaigu, 2140 m elevation, Mt. Beidawushan, Pingtung County, Taiwan (Fig. 2; Table 2; 22°36.93'N, 120°44.53'E), 20 June 2023 by Ting-Yang Chien, Yi-Ting Chung, Zong-Yu He and Jing-Jie Lo. Deposited in Taiwan Agricultural Research Institute (TARI, Wufeng, Taiwan). ***Paratypes***. Paratype 1, an adult male with the femur orange plaque, 36.65 mm (Fig. 8C, D). Collected in type locality 18 June 2024 by Yu-Fang Tsai. Deposited in Taiwan Agricultural Research Institute (TARI, Wufeng, Taiwan). Paratype 2, an adult female, 35.50 mm, (Fig. 8E, F). Collected in type locality 6 June 2024 by Yu-Fang Tsai. Deposited in Taiwan Agricultural Research Institute (TARI, Wufeng, Taiwan). A total of 44 specimens (37 males and 7 females) are designated as paratypes in this study. All paratypes were collected from the type locality. The sampling periods and numbers of specimens collected are as follows: 7 June 2014, 2 males 1 female; 25 June 2018, 8 males 1 female; 27 June 2022, 4 males 1 female; 20 June 2023, 3 males 2 females; 6 June 2024, 3 males 1 female; 9 June 2024, 3 males; 18 June 2024, 9 males; 22 June 2024, 2 males; 23 June 2024, 4 males 1 female. The specimens are deposited in the Taiwan Agricultural Research Institute (TARI), the Biological Museum of the University of Taipei (BMUT), and will be transferred to other museums later.

##### Diagnosis.

A moderately sized species of *Lucanus*. Males measure 36.22 ± 3.69 mm in body length (range: 28.07–46.79 mm; *n =* 38), while females measure 34.53 ± 2.44 mm (range: 30.90–37.17 mm; *n =* 7). This species is morphologically similar to *L. kanoi kanoi*, but males are slightly smaller and females slightly larger. The molecular phylogenetics based on the two mtDNA markers showed an early divergence of the subspecies from *L. kanoi kanoi* (Suppl. material 7).

**Figure 9.**
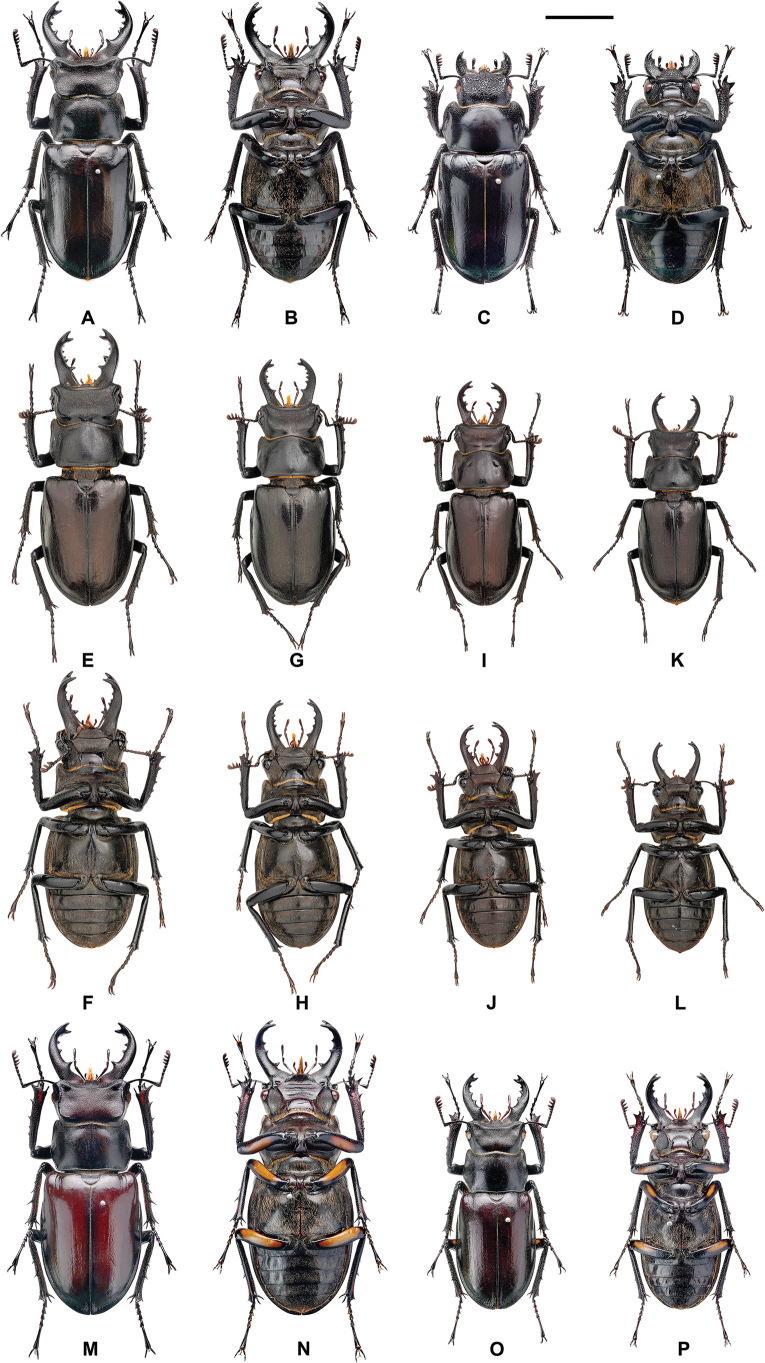
*Lucanus kanoi kavulunganus* subsp. nov., variation in gender, size, mandible, and color. **A, B, E, F**. Maximum form of male; **C, D**. Female; **G–J**. Medium form; **K, L**. Minimum form; **M–P**. With the orange plaque on femur. Scale bar: 1 cm.

**Male**. Clypeolabrum shorter and broader than in *L. kanoi kanoi*, with the labrum clearly separated from the clypeus laterally. Lateral angles of clypeus distinct; frontal ridge indistinct. Elytra glossy, predominantly blackish; a few individuals are reddish brown. Mandibles straight, slightly incurved inward, with a distinct basal tooth positioned close to the base and clearly separated from the adjacent tooth, the basal tooth of the mandible is situated approximately at the basal 1/3–1/4 of its length. Ventral surface between meso- and metacoxae covered with dense, short, yellowish pubescence. Legs blackish to dark brown, matching the coloration of the ventral body surface. A minority of individuals (12/38; 31.6%) exhibit a yellowish to dark orange plaque on the ventral side of the femora, but this trait is inconsistent and not taxonomically informative (Figs 8, 9, Table 3, Suppl. material 1).

**Female**. Externally similar to *L. kanoi kanoi*. Anterior angles of head sharp and distinct. Inner tooth of left mandible single-pointed. Posterior margin of canthus inconspicuous. Head punctation coarse and distinct. Elytra matte, blackish to dark brown. Pronotum not widened at anterior third; evenly rounded anteriorly. Metasternum covered with short pubescence. Femora uniformly dark, lacking the yellowish to orange plaque observed in some males (Figs 8E, 8F, 9C, 9D).

***Genitalia***. Male. In dorsal view, the cephalic process of the paramere is stout. The apical duct and basal belt of the flagellum together measure ~ 1.4 times in length. The ventral plate of the basal piece is well developed and elongate, exhibiting a shallow median concavity and extending distinctly beyond the caudal margin of the basal piece. The ventral plate of the ninth abdominal segment is markedly constricted anterior to the caudal expansion. The flagellum is comparable in length to that of *L. k. kanoi* or *L. piceus*. In lateral view, the apex of the paramere is not emarginate (Fig. 3, Table 3).

Female. Female genitalia do not exhibit significant differences from those of closely related species, including *L. k. kanoi*, *L. piceus*, and *L. ogakii* (Huang and Chen 2010). The spermathecal duct is ~ 2.5× the length of the spermatheca and 1.5× the length of the hemisternite. The spermatheca, which is sclerotized, measures 0.5–0.7× the length of the hemisternite. The spermathecal duct is weakly sclerotized. The spermathecal gland is distinctly broader than the spermatheca and nearly equal in length. The central portion of the ninth tergite is weakly to strongly produced posteriorly. The lateral angles of the terminal abdominal tergite are indistinct, and the terminal abdominal ventrite is medially excavated.

##### Description.

This subspecies, *L. kanoi kavulunganus* is very similar to *L. kanoi kanoi* but slightly smaller. General morphological characteristics, genital anatomy, and color variation are shown in Figs 3, 8, 9, Table 3. The “odd” orange plaque on the femur of male individuals (see description of related taxa in this article) has the highest occurrence rate, with more than 30% of males (31.58%, 12 of 38) exhibiting this characteristic, although this feature is not a reliable basis for taxonomy (Fig. 9, Table 3, Suppl. material 1).

##### Etymology.

The name *kavulunganus* refers to the type locality of this species, Mt. Beidawushan. “Kavulungan” is the name given to Mt. Beidawushan by the indigenous Paiwan people.

##### Distribution.

This small subspecies of *L. kanoi* is currently known to be distributed only in the highland primeval broad-leaved forest areas near Kuaigu on Mt. Beidawushan, with an altitude range of ca 2000 to 2200 meters a.s.l. (Fig. 2, Table 2).

##### Ecology.

Adults are primarily active in June. By comparing the monthly average temperature isotherms for June with the species’ altitudinal range, it is inferred that this stag beetle tends to inhabit cooler environments (Suppl. material 8).

##### Conservation status.

Not evaluated (NE).

##### Remarks.

Based on observations and descriptions from climbers, it is believed that this subspecies is also present on Mt. Nandawushan (Pinayuanan: Itamilimilingan), though confirmation is needed.

#### Lucanus ogakii

Taxon classificationAnimaliaColeopteraLucanidae

Imanishi, 1990

FBF0CC70-D01F-5F9E-BB25-85AC11AD0F53

Fig. 10

Lucanus ogakii Imanishi, 1990: 15–18, figs 1, 2, 2a, 3. Chang 1993: 54, pl. 24, figs I7–1–I4; Mizunuma and Nagai 1994: 214, pl. 12, figs 101–1–5; Chang 2006: 48–49; Fujita 2010: 93–94, pl. 35, fig. 236–1–4; Huang 2016: 128–131; Chang 2024: 46–47.
Lucanus masumotoi Hirasawa & Akiyama, 1990: 53–58, pl. 5 figs 9–11, pl. 6 figs 14, 16, pl. 6 figs 14, 16. Wang 1994: 40–43.
Lucanus kanoi ogakii Imanishi, 1990: 15–18, figs 1, 2, 2a, 3. Huang and Chen 2010: 129–132, pl. 3 figs 22–26, pl. 4 fig. 62, pl. 15 fig. 5, pl. 29 fig. 3, pl. 40 fig. 4, pl. 51 fig. 11, pl. 55 fig. 4.

##### Subspecies.

*Lucanus ogakii chuyunshanus* (Sakaino & Yu, 1993): 15–16, figs 7, 8. Tsai and Yeh 2016: p.3, 8, 9, 12, 16.

*= Lucanus masumotoi chuyunshanus* Sakaino & Yu, 1993: 15–16, figs 7, 8.

##### Material examined.

**Figure 10.**
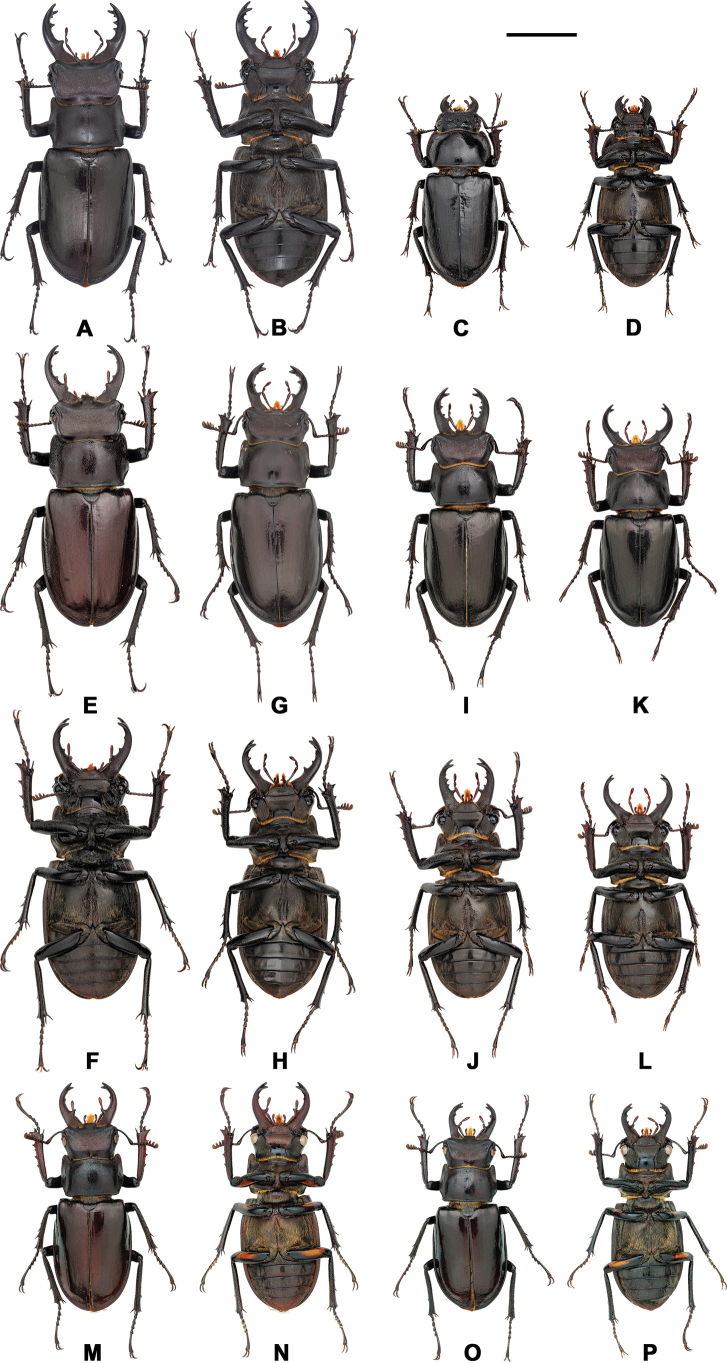
*Lucanus ogakii*, variation in gender, size, mandible, and color. **A, B, E, F**. Maximum form of male; **C, D**. Female; **G–J**. Medium form; **K, L**. Minimum form; **M–P**. With the orange plaque on femur. Scale bar: 1 cm.

A total of 254 males (33.39 ± 3.34 mm, min-max 25.04–42.53 mm) and 41 females (30.82 ± 3.48 mm, min-max 23.93–38.99 mm) specimens collected from 6 localities were examined in this study including 9 individuals (8 males and 1 female) from the type locality (Fig. 2, Table 2). Variation of body size is presented in Fig. 10 and Table 3.

##### Diagnosis.

A moderate to small-sized *Lucanus*, with a male body length averaging 33.39 ± 3.34 mm (range: 25.04–42.53 mm, *n =* 254), and a female body length averaging 30.82 ± 3.48 mm (range: 23.93–38.99 mm, *n =* 41). This taxon is similar to *L. k. kanoi* and *L. piceus* but generally smaller. The species is blackish in color and is distributed on the eastern side of the central mountain range, from Hualien to Taitung County.

##### Description.

Body length. Male: 24.53–45.72 mm; Female: 23.93–38.99 mm (Kurosawa 1966; Chang 2006; Huang and Chen 2010; Chang 2024; this study; Table 3). Most individuals exhibit dark, blackish body coloration, although reddish individuals are also present. The legs and femora of males are black, with a very rare occurrence of a yellowish to orange plaque on the femur (Huang 2016). The color, external morphology, and anatomy of the genitalia are shown in Figs 3, 10, and Table 3.

A very rare characteristic (< 2%, 3 of 246 specimens in this study) is the presence of a yellowish to orange plaque on the femur of males, but this feature is not a stable morphological trait and cannot be used for taxonomic classification. Therefore, this plaque can only be regarded as a trait variation within the species and its related taxa (Fig. 10, Table 3, Suppl. material 1).

##### Distribution.

This species inhabits primary broad-leaved forests at mid-to-high altitudes between 1960 and 2564 meters, and is distributed on the eastern side of the central mountain range, from Hualien to Taitung County (Fig. 2, Table 2).

##### Ecology.

Adults are primarily active from late May to June. By comparing the monthly average temperature isotherms for June with the species’ altitudinal range, it is inferred that this stag beetle tends to inhabit cooler environments (Suppl. material 8).

##### Conservation status.

Not evaluated (NE).

##### Remarks.

In the original article on the subspecies *L. ogakii chuyunshanus*, Sakaino and Yu (1993) emphasized that the major distinguishing characteristic between *L. ogakii* (then referred to as *L. masumotoi*) and this subspecies was the yellowish plaque on the femur (Sakaino and Yu 1993). However, the two male specimens of *L. ogakii chuyunshanus* examined in MSME do not possess this characteristic (Suppl. material 4B, D). Therefore, neither of these two male specimens of *L. ogakii chuyunshanus* that lack original labels can be regarded as the type specimen designated in the original article by Sakaino and Yu (1993).

The *Lucanus kanoi* species complex examined in this study can be distinguished using the dichotomous key provided below. This key is based on external characters of the type specimens of *L. kanoi* (*L. k. kanoi*, sensu stricto in this study), *L. piceus* stat. nov. (formerly *L. k. piceus*), and *L. kanoi kavulunganus* subsp. nov. Additional examined specimens and their diagnostic characters are summarized in Table 3 and presented in Suppl. materials 1, 3.

### Key to the males of the *Lucanus kanoi* species complex

**Table:** 

1	Mandibles with a single internal tooth; paramere slender; elytra broad and rounded, often with a distinct reddish tint; femora rarely bearing yellowish-brown stripes on the underside	***Lucanus ogakii***
–	Mandibles with two or more denticles/teeth; paramere stout; elytra blackish to reddish-brown, not distinctly rounded	**2**
2	Body piceous to blackish; elytra more nitidous, with finer and sparser punctures; legs uniformly dark; mandibles generally straighter with basal tooth not very close to the base	***Lucanus piceus* stat. nov**.
–	Body blackish, reddish-brown, or chocolate-brown; elytra glabrous to slightly pubescent, usually less shining; femora occasionally with yellow to orange plaque; mandibles shorter, robust, and strongly arcuate, with the basal tooth situated close to the base	**3**
3	Clypeus with well-defined lateral angles; labrum clearly separated from clypeus at the sides; elytra typically glossy; distribution in southern Taiwan mountains	***Lucanus kanoi kavulunganus* subsp. nov**.
–	Clypeus without distinct lateral angles (labrum not clearly separated; lateral/frontal ridges not markedly protruding); elytra glabrous to sparsely pubescent and less nitidous; mandibles short, strongly arcuate, with basal tooth very close to the base; distribution in central to northern Taiwan	***Lucanus kanoi kanoi***

### Morphometric analysis

**Figure 11.**
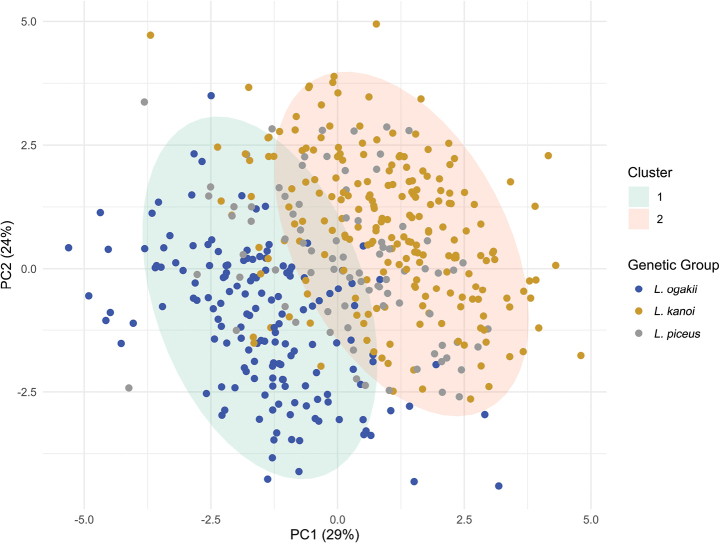
Assignment of individuals based on the two-cluster solution from morphological clustering analysis. Cluster 1 (*n* = 202) included the majority of *L. ogakii* specimens (91.6%, *n* = 142), while Cluster 2 (*n* = 247) comprised most *L. kanoi* (85.2%, *n* = 178) and *L. piceus* (65.9%, *n* = 56) specimens.

**Figure 12.**
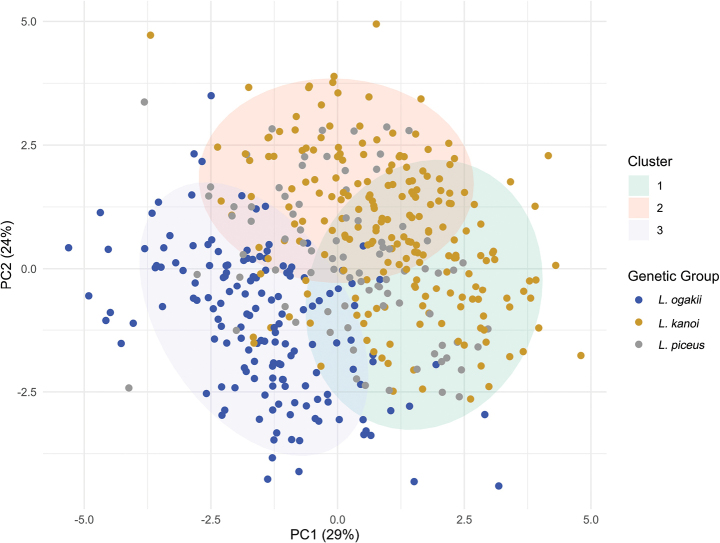
Assignment of individuals based on the three-cluster solution from morphological clustering analysis. Cluster 1 included 151 individuals, Cluster 2 had 139, and Cluster 3 comprised 129 individuals. Most *L. ogakii* specimens (82.6%, *n* = 128) were assigned to Cluster 1, while the majority of *L. kanoi* (94.3%, *n* = 197) and *L. piceus* (87.1%, *n* = 74) specimens were distributed across Clusters 2 and 3.

A total of 449 individuals from three genetically defined taxa were analyzed: *L. ogakii* (*n =* 155), *L. kanoi* (*n =* 209), and *L. piceus* (*n =* 85). Gap statistical analysis identified two clusters as the optimal solution and three clusters as a suboptimal solution (Suppl. material 9). Based on the two-cluster solution, individuals were assigned to Cluster 1 (*n =* 202) or Cluster 2 (*n =* 247). Among the *L. ogakii* specimens, 142 (91.6%) were assigned to Cluster 1. In contrast, 178 (85.2%) of *L. kanoi* and 56 (65.9%) of *L. piceus* individuals were assigned to Cluster 2 (Fig. 11). Under the three-cluster solution, Cluster 1 included 151 individuals, Cluster 2 had 139, and Cluster 3 comprised 129 individuals. Most *L. ogakii* individuals (128, 82.6%) were grouped into Cluster 1. The majority of *L. kanoi* (197, 94.3%) and *L. piceus* (74, 87.1%) individuals were distributed across Clusters 2 and 3 (Fig. 12).

### Biogeography and phenology

The biogeographical distributions of *L. k. kanoi*, *L. piceus*, and *L. ogakii* on Taiwan Island are clearly distinct and do not overlap, meaning these taxa do not coexist in the same geographical space. Even in northern Taiwan, where the distributions of *L. k. kanoi* and *L. piceus* occur in close horizontal proximity, there is currently no evidence of sympatry between the two species. (Fig. 2, Table 2). Based on the current morphological and molecular evidence and the descriptions in the original articles, taxa collected from central Taiwan, such as Nantou County, are re-identified as *L. k. kanoi*, while taxa collected from northern or northeastern Taiwan, including Taoyuan City, New Taipei City, and Yilan County, are re-identified as *L. piceus*. The new subspecies of *L. kanoi* described herein, *L. k. kavulunganus*, is restricted to Mt. Beidawushan in southern Taiwan. In contrast, taxa distributed in eastern and southeastern Taiwan belong to *L. ogakii*. However, the subspecies *L. ogakii chuyunshanus* was described from Mt. Chuyunshan on the western side of the Central Mountain Range. Although no specimens of *L. o. chuyunshanus* from this type locality are currently available, and we were unable to examine any such material from either museums or private collections during the course of this study, most publications have regarded the *L. ogakii* specimens from Siangyang, Taitung, as belonging to *L. o. chuyunshanus* (Tsai and Yeh 2016; Chang 2024). The three taxa, *L. k. kanoi*, *L. piceus*, and *L. ogakii*, together with the two subspecies, *L. k. kavulunganus* and *L. o. chuyunshanus*, inhabit mid- to high-elevation broadleaf forests, with *L. ogakii* occurring at the highest elevations, up to 2300 m (Fig. 2, Table 2).

Adults of *L. k. kanoi kanoi* and *L. piceus* are most frequently observed in June, with a limited number persisting into July. Records from August are scarce, with only a few individuals observed annually. In northern and central Hualien (e.g., Bilu Sacred Tree and Ruisui), *L. ogakii* has mainly been observed from June to early July. Although the occurrence period of the Mt. Chuyunshan population (the type locality of *L. o. chuyunshanus*) remains unknown, the Siangyang population has primarily been recorded from early to mid-June. June represents the peak occurrence period for all three taxa, including the newly described subspecies *L. kanoi kavulunganus*. Overlaying the June average temperature isotherms with the known distribution ranges of these taxa indicates a clear preference for cooler, high-altitude environments. Given that these insects are nocturnal, and that mountainous regions above 2,000 m in Taiwan typically experience diurnal temperature fluctuations of approximately 7–8 °C during June (Chi 1970), it is plausible that their actual thermal preferences are lower than those suggested by current phenological and distributional records (Suppl. material 8).

## Discussion

### Systematic status of *L. kanoi*, *L. piceus*, and *L. ogakii*

Historically, there has been significant debate regarding the taxonomic status of *L. kanoi kanoi* and *L. piceus*. Many authors have argued against retaining *L. k. piceus* as a subspecies, citing a lack of sufficient evidence to distinguish *L. k. kanoi* from *L. k. piceus*, and have advocated for considering *L. k. kanoi* as a single species without relative subspecies at the taxonomic level (Huang 2016; Chang 2024). This argument is based on two main points: 1) The geographical distributions of these two taxa overlap, making it difficult to satisfy the prerequisite of geographic isolation required for subspecies designation. 2) The external morphological characteristics of these two taxa, such as body color, exhibit a vast range of intraspecific variation that exceeds the interspecific variation. For example, *L. k. kanoi* does not consistently exhibit a reddish-brown body color; many black individuals have been observed and recorded. Similarly, *L. k. piceus* does not consistently exhibit a dark blackish body color, as originally described, with numerous reddish-brown individuals observed. These morphological and color variations are also demonstrated in Figs 6, 7, and Table 3 of this study.

In some earlier literature, such as Wang (1990, 1994), the subspecies *L. k. piceus* was not mentioned or discussed at all. Two possibilities for this omission are speculated: first, Wang may not have examined the original work by Kurosawa (1966) and thus was unaware of his descriptions and taxonomic judgments of *L. k. kanoi* and *L. k. piceus*. The second possibility is that Wang did not consider *L. k. piceus* taxonomically significant and deliberately ignored it in his work (Wang 1990, 1994).

Another viewpoint maintains that both subspecies, *L. k. kanoi* and *L. k. piceus*, should be retained. This perspective suggests that *L. ogakii* should be subsumed into *L. kanoi* as a southeast subspecies, *L. k. ogakii* (Huang and Chen 2010). This argument is based on the lack of clear anatomical differences in the male genitalia characters between *L. kanoi*, *L. piceus*, and *L. ogakii*. As a result, it is suggested that these three taxa have not reached a level of differentiation significant enough to justify their recognition as separate species. The recommendation is to classify these three taxa as subspecies under *L. kanoi*. This opinion aligns with the biological species concept (BSC) (Mayr and Ashlock 1991), which posits that species are reproductively isolated. Under the framework of BSC, the evidence presented by Huang and Chen (2010) is sufficient and credible for species delimitation. However, our molecular phylogenetic results indicate that the three taxa represent three distinct evolutionary lineages and should therefore be treated as separate species. *Lucanus ogakii* forms a lineage clearly distinct from the *L. kanoi*–*L. piceus* ingroup, whereas *L. kanoi* and *L. piceus* constitute a pair of sister species characterized by a short internal branch in the phylogenetic topology. (Fig. 5, Suppl. material 6). Despite *L. piceus* being designated as a subspecies of *L. kanoi* in the original literature, our results affirm that *L. kanoi* and *L. piceus* should be treated as separate species. This conclusion is supported by the phylogenetic species concept (Coyne and Orr 2004), which defines species based on their evolutionary relationships. Therefore, these three taxa should be regarded as three distinct species, with *L. ogakii* representing an earlier-diverging lineage from the *L. kanoi*–*L. piceus* clade, while *L. kanoi* and *L. piceus* are sister species that underwent more recent speciation within the group (Fig. 5, Suppl. material 6).

The divergence between *L. k. kanoi* and *L. piceus* likely occurred relatively recently. Genetic distances between specimens from the type localities are approximately 2.48% for the CO1 gene and 1.13% for the Wnt gene (SJ–SG, Suppl. materials 10, 11). According to our molecular evidence, the divergence between *L. k. kanoi* and *L. piceus* is estimated at ca 1.25 Mya (95% HPD = 0.82–1.84 Mya), whereas *L. ogakii* diverged from the *L. k. kanoi*–*L. piceus* lineage at ca 3.65 Mya (95% HPD = 2.55–5.05 Mya), suggesting that *L. ogakii* represents an earlier-diverging lineage within the complex (Suppl. material 7). However, further studies are required to refine the divergence model and to better understand the patterns of genetic differentiation between these taxa.

### Biogeographical distribution of *L. kanoi*, *L. piceus*, and *L. ogakii*

Although the distributions of *L. k. kanoi* and *L. piceus* do not overlap, some populations of these two taxa are geographically proximate in the northern mountains of Taiwan (Fig. 2). Despite the taxa being originally described and designated as a paratype of *L. k. piceus* in Kurosawa (1966), molecular genetic analysis of the *Lucanus* species from Mt. Lalashan and its neighboring Mt. Tamanshan in Taoyuan reveals that these populations belong to *L. k. kanoi* (Fig. 5, Table 2, Suppl. material 6). Further south, at Mt. Lidongshan in Hsinchu County, *L. k. kanoi* is also found (Figs 2, 5, Table 2, Suppl. material 6). In contrast, at more southerly locations such as Mt. Tielikushan, Yuanyang Lake, and Shengguang, the *Lucanus* beetles are genetically classified as *L. piceus* (Figs 2, 5, Table 2, Suppl. material 6). Therefore, in the Taoyuan–Hsinchu area, the geographical distributions of these two taxa are closely adjacent but do not overlap or occur in sympatry (Fig. 2).

Molecular results from this study confirm that *L. k. kanoi* and *L. piceus* represent two separate species, with distinct short-branch monophyletic clades. While their geographical distributions do not overlap, the observed closely adjacent pattern of their distributions in northern Taiwan remains puzzling. This phenomenon is an intriguing issue that warrants further investigation. Our current evidence confirms the existence of this odd distribution pattern but does not provide an explanation for the historical events that have led to this distribution.

We propose two potential hypotheses that may guide future research:

1. Northward Spread Hypothesis: The northern *L. k. kanoi* populations found in Mt. Lalashan, including adjacent populations in Mt. Tamanshan and Mt. Lidongshan, may have originated from a central Taiwan population. These populations could have expanded northward and established a founder population in the northern exclave. In other words, this northern *L. k. kanoi* population could represent an enclave of *L. piceus*’ territory.
2. Hybridization Hypothesis: Another possibility is that the *L. k. kanoi* populations in northern Taiwan are the result of hybridization between ancestral maternal *L. k. kanoi* and paternal *L. piceus*. Given that the molecular markers used in this study primarily relied on mitochondrial gene fragments (with one nuclear gene fragment), the results may reflect a biased view of maternal inheritance. This hypothesis warrants further testing, such as using SNP-based technologies to examine potential genetic differentiation between *L. k. kanoi* and *L. piceus* and explore the evolutionary history of ancestral gene flow.

An earlier study by Tsai and Yeh (2016) aimed to explore the geographical distribution, genetic relationships, and genetic boundaries of *L. k. kanoi*, *L. piceus*, and *L. ogakii*, but it was limited by several issues. First, the classification of *L. k. kanoi* from Mt. Lalashan as *L. piceus* was incorrect due to the lack of a proper phylogenetic analysis. This misclassification is evident in the species distribution map provided in that study. Second, the study’s distribution map was not based on actual specimens but instead relied on speculative estimates, resulting in discrepancies between their findings and the empirical specimen data presented here. In reality, the boundary between *L. k. kanoi* and *L. piceus* is more complex than previously thought, with a closely adjacent area at Mt. Lalashan and Mt. Tamanshan. Our study, based on evidence from actual specimens, provides a more accurate depiction of the geographical distribution and boundaries of these two taxa (Fig. 2). The question of why these two species, which are clearly differentiated in their phylogenetic relationships, exhibit such a complex distribution in northern Taiwan remains unanswered. Further investigation into their population genetics through genomic studies is required to shed light on the cause of this unusual distribution pattern.

### Mt. Beidawushan: the southern exclave of *L. kanoi*

This study uncovers a small, isolated population of *L. kanoi* located at the southern end of Taiwan Island. This population, first discovered by THH in 2010, is currently known to inhabit only the Mt. Beidawushan areas (Fig. 2). Due to its proximity to other populations, it was initially thought that this group might belong to the southern population of *L. ogakii*, or possibly represent the subspecies *L. o. chuyunshanus*. However, molecular genetic evidence presented in this study clearly indicates that this population, residing in the Mt. Beidawushan area, is a subclade of *L. kanoi* and does not belong to the *L. ogakii* lineage.

The origin of the small *L. kanoi* population in Mt. Beidawushan and the absence of intermediate populations between this enclave and the larger central population raise intriguing questions. The southernmost continuous population of *L. k. kanoi* occurs along the Shalisian Logging Trail, approximately 115 km north of Mt. Beidawushan (Fig. 2, Table 2). Interestingly, despite the presence of continuous montane broadleaf forest above 1600 m between these two localities, no populations of *L. kanoi* have been recorded in this intervening region, resulting in a distinct exclave at the southern tip of the island, a so-called “sky island” of Taiwan. This isolated population represents a noteworthy subject for further investigation.

Preliminary analyses of genetic differentiation among the northern, central, and southern populations of *L. kanoi* (including the newly described subspecies *L. k. kavulunganus*) reveal that genetic distances between the northern and central populations (LLS–SG) are approximately 0.78% for the CO1 gene and 0.45% for the Wnt gene, whereas those between the central and southern populations (SG–BDW) are approximately 1.4% for the CO1 gene and 0.91% for the Wnt gene (Suppl. materials 10, 11). These results indicate that the northern and central populations are genetically very similar, exhibiting minimal differentiation, while the Mt. Beidawushan population likely diverged from the central population relatively recently. This finding further supports the inference that the southern population represents a recently isolated lineage at the southernmost extent of the species’ distribution. Notably, divergence time estimates based on mitochondrial markers CO1 and 16S suggest that northern and central populations of *L. k. kanoi* diverged from the southern population, herein referred to as the subspecies *L. k. kavulunganus* from Mt. Beidawushan, ca 0.52 Mya (95% HPD = 0.26–0.88 Mya; Suppl. material 7).

### On the taxonomic status of *Lucanus ogakii chuyunshanus*

Although some literature has suggested that the southern subspecies of *L. ogakii*, namely *L. ogakii chuyunshanus*, should no longer be retained and its taxonomic validity should be dismissed (Chang 2024), this study sets aside discussion on the status or validity of this subspecies. We argue that this issue can only be meaningfully addressed once further evidence becomes available, or at least when specimens originating from the type locality, Mt. Chuyunshan, are obtained for examination.

We highlight several considerations that should be taken into account in future evaluations of this taxon:

1. Loss of the type specimen. The only known type specimen (holotype) of *L. ogakii chuyunshanus* has been lost and is no longer available for examination. Moreover, no specimens of this taxon have been discovered at the type locality, Mt. Chuyunshan, either in museum or private collections.
2. Morphological and taxonomic considerations. In the original description, *L. o. chuyunshanus* was distinguished from *L. ogakii* by a more reddish body coloration and the presence of a yellowish to orange plaque on the femur. However, these traits are neither stable nor sufficient for reliable taxonomic distinction within the *L. kanoi* species complex. Our study provides detailed evidence (Figs 6, 7, 8, 9, 10, Table 3) showing that such features occur in fewer than 3% of male *L. ogakii* individuals, rendering them too rare to be taxonomically informative. While Sakaino and Yu (1993) described *L. ogakii* with this rare trait as a subspecies with special characters, the taxonomic relevance of this feature remains questionable.
3. Representativeness of the Siangyang population. Whether the *L. ogakii* population from Siangyang can represent *L. o. chuyunshanus* remains uncertain. Although many studies have treated the Siangyang population as belonging to *L. o. chuyunshanus* (Tsai and Yeh 2016; Chang 2024), the overall distribution records of *L. ogakii* in Taiwan are restricted to the eastern slopes of the Central Mountain Range (Fig. 2, Table 2). In contrast, the type locality, Mt. Chuyunshan, is located on the western side of the range, where no *L. ogakii* populations are currently known.
4. Biogeography. Building upon point 3, while field surveys at Mt. Chuyunshan have not been possible for the past two decades due to natural disturbances, the Siangyang population has traditionally been considered part of *L. o. chuyunshanus* (Tsai and Yeh 2016; Chang 2024). However, the Siangyang and Ruisui populations are separated by only ~42 km, with continuous mid-elevation broadleaf forest between them. Thus, no habitat gap or geographical isolation exists, contradicting the prerequisite of geographic separation necessary for subspecies recognition.
5. Molecular phylogeny and genetic structure. Despite the relatively short geographic distance (~ 42 km) between Siangyang and Ruisui, our molecular phylogenetic analyses indicate a certain degree of differentiation between these populations. Specifically, the genetic distances between the Siangyang and Ruisui populations exceed those observed between the Bilu and Ruisui populations (Figs 2, 5, Table 2, Suppl. materials 6, 7, 10, 11).

Until these issues are more thoroughly addressed, the validity of *L. ogakii chuyunshanus* as a subspecies cannot be clearly resolved. Future research, with more comprehensive evidence, will be required to reassess the taxonomic status of this subspecies and provide a more convincing resolution.

### Species complexes as a persistent challenge in taxonomy

Based on the established relationship between ontogeny and phylogeny, it is well recognized that organisms within a lineage may exhibit divergent growth trajectories, a phenomenon referred to as heterochrony (Alberch et al. 1979; Klingenberg 1998). Such heterochronic variation is often species-specific and can therefore be informative for taxonomic delineation. Given the difficulty in distinguishing species within the *L. kanoi* species complex based solely on external morphological features (Huang and Chen 2010), the present study incorporates analyses of allometric growth patterns between taxa to identify diagnostic morphological traits. The present analysis effectively separates *L. ogakii* from the *L. kanoi*–*L. piceus* lineage within the species complex (Fig. 11), thereby refining the earlier taxonomic treatment of Huang and Chen (2010), who considered all three taxa conspecific.

In regions where the distributions of *L. k. kanoi* and *L. piceus* are geographically adjacent, such as the montane areas of Taoyuan in northern Taiwan, one or multiple hybridization events may have occurred. Such hybridization among closely related species can reduce the diagnostic power of morphological evidence, as genetic introgression may produce a “fusion effect” that blurs species boundaries between already diverged taxa (Stebbins 1959; Bullini 1994; Arnold 1997). This possibility is further supported by Tsai and Yeh (2016), who suggested that hybridization and partial gene flow may exist between *L. k. kanoi* and *L. piceus*. Consequently, although our phylogenetic analyses recovered *L. k. kanoi* and *L. piceus* as two clearly distinguishable short-branched lineages, these taxa still share considerable similarities in both external morphology and genetic characteristics.

For species complexes, both systematic classification and phylogenetic inference remain highly challenging, as researchers must contend with factors such as potential hybridization and gene flow among closely related taxa, as well as morphological similarity that obscures taxonomic resolution. In such cases, molecular evidence and the recognition of monophyletic lineages under the phylogenetic species concept (PSC) provide an objective framework, as demonstrated by the results of this study.

## Conclusions

This study resolves the systematic relationships among *Lucanus kanoi*, *L. piceus*, and *L. ogakii*, supporting their recognition as three distinct species. A new subspecies, *L. kanoi kavulunganus* subsp. nov., is described based on its geographical isolation and morphological characters. The geographic distributions of these taxa are also thoroughly examined and clearly delineated.

### Future study prospects

Despite recent advances, key questions concerning the evolutionary history and systematics of the *Lucanus kanoi* species complex remain unanswered, highlighting the need for further investigation. Key questions to be addressed in subsequent studies include the historical dispersal pathways of each population, the potential for hybridization among the current species-level taxa, the evolutionary status and divergence patterns between the *L. kanoi* species complex and the *L. taiwanus* clade, as well as the conditions of population expansion and contraction throughout their lineage history. Moreover, the dynamics of intra-species and inter-species gene flow remain crucial areas for investigation.

To address these questions, future research will require the application of more advanced molecular genetic techniques, such as ddRAD sequencing, to provide deeper phylogenetic resolution. Similar approaches have been successfully applied in recent studies of other Taiwanese stag beetles and coleopteran taxa (e.g., De Vivo et al. 2023; Huang et al. 2024), demonstrating the effectiveness of molecular data in resolving taxonomic ambiguities. These studies offer valuable methodological references for ongoing and future work on the *Lucanus* species complex.

In parallel, more sophisticated and precise geometric morphometric techniques and comparative frameworks, such as the geometric morphometric approaches and analytical models proposed by Tong et al. (2021) will also be integrated. These approaches are expected to improve the resolution of morphological analyses and offer more robust solutions to the taxonomic challenges identified in this study.

## Supplementary Material

XML Treatment for Lucanus kanoi kanoi

XML Treatment for Lucanus piceus

XML Treatment for Lucanus kanoi kavulunganus

XML Treatment for Lucanus ogakii

## Additional information

### Use of AI

No use of AI was reported.

### Author ORCIDs

Shu-Ping Wu https://orcid.org/0000-0001-6764-1118

Yu-Fang Tsai https://orcid.org/0000-0002-0336-696X

Chung-Chi Hwang https://orcid.org/0000-0002-4780-4909

### Data availability

All of the data that support the findings of this study are available in the main text or Supplementary Information.

## Supplementary materials

Supplementary material 1 Examined sample size and morphological data of this study Data type docx https://binary.pensoft.net/file/1517888This dataset is made available under the Open Database License (http://opendatacommons.org/licenses/odbl/1.0/). The Open Database License (ODbL) is a license agreement intended to allow users to freely share, modify, and use this Dataset while maintaining this same freedom for others, provided that the original source and author(s) are credited. Shu-Ping Wu, Yu-Fang Tsai, Ting-Yang Chien, Yi-Ting Chung, Ching-Jung Lai, Tsung-Hsien Hou, Chung-Chi Hwang

Supplementary material 2 Permission numbers for scientific study of government agencies Data type docx https://binary.pensoft.net/file/1517889This dataset is made available under the Open Database License (http://opendatacommons.org/licenses/odbl/1.0/). The Open Database License (ODbL) is a license agreement intended to allow users to freely share, modify, and use this Dataset while maintaining this same freedom for others, provided that the original source and author(s) are credited. Shu-Ping Wu, Yu-Fang Tsai, Ting-Yang Chien, Yi-Ting Chung, Ching-Jung Lai, Tsung-Hsien Hou, Chung-Chi Hwang

Supplementary material 3 The acronyms of museums included in this study Data type docx https://binary.pensoft.net/file/1517890This dataset is made available under the Open Database License (http://opendatacommons.org/licenses/odbl/1.0/). The Open Database License (ODbL) is a license agreement intended to allow users to freely share, modify, and use this Dataset while maintaining this same freedom for others, provided that the original source and author(s) are credited. Shu-Ping Wu, Yu-Fang Tsai, Ting-Yang Chien, Yi-Ting Chung, Ching-Jung Lai, Tsung-Hsien Hou, Chung-Chi Hwang

Supplementary material 4 Collectibles of “*L. o. chuyunshanus*” from MSME https://doi.org/10.3897/zookeys.1267.160494.suppl4 Data type docx https://binary.pensoft.net/file/1517891This dataset is made available under the Open Database License (http://opendatacommons.org/licenses/odbl/1.0/). The Open Database License (ODbL) is a license agreement intended to allow users to freely share, modify, and use this Dataset while maintaining this same freedom for others, provided that the original source and author(s) are credited. Shu-Ping Wu, Yu-Fang Tsai, Ting-Yang Chien, Yi-Ting Chung, Ching-Jung Lai, Tsung-Hsien Hou, Chung-Chi Hwang

Supplementary material 5 GenBank accession numbers of DNA sequences used in this study Data type docx https://binary.pensoft.net/file/1517892This dataset is made available under the Open Database License (http://opendatacommons.org/licenses/odbl/1.0/). The Open Database License (ODbL) is a license agreement intended to allow users to freely share, modify, and use this Dataset while maintaining this same freedom for others, provided that the original source and author(s) are credited. Shu-Ping Wu, Yu-Fang Tsai, Ting-Yang Chien, Yi-Ting Chung, Ching-Jung Lai, Tsung-Hsien Hou, Chung-Chi Hwang

Supplementary material 6 Bayesian phylogenetic relationships of the *Lucanus kanoi* species complex Data type docx https://binary.pensoft.net/file/1517893This dataset is made available under the Open Database License (http://opendatacommons.org/licenses/odbl/1.0/). The Open Database License (ODbL) is a license agreement intended to allow users to freely share, modify, and use this Dataset while maintaining this same freedom for others, provided that the original source and author(s) are credited. Shu-Ping Wu, Yu-Fang Tsai, Ting-Yang Chien, Yi-Ting Chung, Ching-Jung Lai, Tsung-Hsien Hou, Chung-Chi Hwang

Supplementary material 7 Tree of divergence time estimated with BEAST Data type docx https://binary.pensoft.net/file/1517894This dataset is made available under the Open Database License (http://opendatacommons.org/licenses/odbl/1.0/). The Open Database License (ODbL) is a license agreement intended to allow users to freely share, modify, and use this Dataset while maintaining this same freedom for others, provided that the original source and author(s) are credited. Shu-Ping Wu, Yu-Fang Tsai, Ting-Yang Chien, Yi-Ting Chung, Ching-Jung Lai, Tsung-Hsien Hou, Chung-Chi Hwang

Supplementary material 8 Geographical distribution of the *L. kanoi* species complex in Taiwan Data type docx https://binary.pensoft.net/file/1517895This dataset is made available under the Open Database License (http://opendatacommons.org/licenses/odbl/1.0/). The Open Database License (ODbL) is a license agreement intended to allow users to freely share, modify, and use this Dataset while maintaining this same freedom for others, provided that the original source and author(s) are credited. Shu-Ping Wu, Yu-Fang Tsai, Ting-Yang Chien, Yi-Ting Chung, Ching-Jung Lai, Tsung-Hsien Hou, Chung-Chi Hwang

Supplementary material 9 Gap statistic analysis identified two clusters as the optimal solution and three clusters as a suboptimal solution for external morphological data Data type docx https://binary.pensoft.net/file/1517896This dataset is made available under the Open Database License (http://opendatacommons.org/licenses/odbl/1.0/). The Open Database License (ODbL) is a license agreement intended to allow users to freely share, modify, and use this Dataset while maintaining this same freedom for others, provided that the original source and author(s) are credited. Shu-Ping Wu, Yu-Fang Tsai, Ting-Yang Chien, Yi-Ting Chung, Ching-Jung Lai, Tsung-Hsien Hou, Chung-Chi Hwang

Supplementary material 10 Genetic distances among *L. kanoi* species complex and three outgroup taxa in CO 1 gene (below diagonal) and 16S rRNA gene (above diagonal) Data type docx https://binary.pensoft.net/file/1517897This dataset is made available under the Open Database License (http://opendatacommons.org/licenses/odbl/1.0/). The Open Database License (ODbL) is a license agreement intended to allow users to freely share, modify, and use this Dataset while maintaining this same freedom for others, provided that the original source and author(s) are credited. Shu-Ping Wu, Yu-Fang Tsai, Ting-Yang Chien, Yi-Ting Chung, Ching-Jung Lai, Tsung-Hsien Hou, Chung-Chi Hwang

Supplementary material 11 Genetic distances among *L. kanoi* species complex and three outgroup taxa in Wnt gene Data type docx https://binary.pensoft.net/file/1517898This dataset is made available under the Open Database License (http://opendatacommons.org/licenses/odbl/1.0/). The Open Database License (ODbL) is a license agreement intended to allow users to freely share, modify, and use this Dataset while maintaining this same freedom for others, provided that the original source and author(s) are credited. Shu-Ping Wu, Yu-Fang Tsai, Ting-Yang Chien, Yi-Ting Chung, Ching-Jung Lai, Tsung-Hsien Hou, Chung-Chi Hwang

Supplementary material 12 Assignment of individuals based on the three-cluster solution from morphological clustering analysis Data type docx https://binary.pensoft.net/file/1517899This dataset is made available under the Open Database License (http://opendatacommons.org/licenses/odbl/1.0/). The Open Database License (ODbL) is a license agreement intended to allow users to freely share, modify, and use this Dataset while maintaining this same freedom for others, provided that the original source and author(s) are credited. Shu-Ping Wu, Yu-Fang Tsai, Ting-Yang Chien, Yi-Ting Chung, Ching-Jung Lai, Tsung-Hsien Hou, Chung-Chi Hwang

Supplementary material 13 Genetic distances among *L. kanoi* species complex and three outgroup taxa in CO 1 gene (below diagonal) and 16S rRNA gene (above diagonal) Data type docx https://binary.pensoft.net/file/1517900This dataset is made available under the Open Database License (http://opendatacommons.org/licenses/odbl/1.0/). The Open Database License (ODbL) is a license agreement intended to allow users to freely share, modify, and use this Dataset while maintaining this same freedom for others, provided that the original source and author(s) are credited. Shu-Ping Wu, Yu-Fang Tsai, Ting-Yang Chien, Yi-Ting Chung, Ching-Jung Lai, Tsung-Hsien Hou, Chung-Chi Hwang

Supplementary material 14 Genetic distances among *L. kanoi* species complex and three outgroup taxa in Wnt gene Data type docx https://binary.pensoft.net/file/1517901This dataset is made available under the Open Database License (http://opendatacommons.org/licenses/odbl/1.0/). The Open Database License (ODbL) is a license agreement intended to allow users to freely share, modify, and use this Dataset while maintaining this same freedom for others, provided that the original source and author(s) are credited. Shu-Ping Wu, Yu-Fang Tsai, Ting-Yang Chien, Yi-Ting Chung, Ching-Jung Lai, Tsung-Hsien Hou, Chung-Chi Hwang

## References

[B1] Alberch P, Gould SJ, Oster GF, Wake DB (1979) Size and shape in ontogeny and phylogeny. Paleobiology 5(3): 296–317. 10.1017/S0094837300006588

[B2] Arnold ML (1997) Natural hybridization and evolution. Oxford University Press, NY, USA. 232 pp. 10.1093/oso/9780195099744.001.0001

[B3] Bouckaert R, Heled J, Kühnert D, Vaughan T, Wu CH, Xie D, Suchard MA, Rambaut A, Drummond AJ (2014) BEAST 2: A software platform for Bayesian evolutionary analysis. PLoS Computational Biology 10(4): e1003537. 10.1371/journal.pcbi.1003537 PMC3985171 24722319

[B4] Bullini L (1994) Origin and evolution of animal hybrid species. Trends in Ecology & Evolution 9(11): 422–426. 10.1016/0169-5347(94)90124-4 21236911

[B5] Chang YJ (1993) Stag Beetles of Taiwan. Newton Publishing Co. Ltd., Taipei, 111 pp. [in Chinese]

[B6] Chang YJ (2006) Stag Beetles 54. Yuan-Liou Publishing Co., Ltd., Taipei, 160 pp. [in Chinese]

[B7] Chang YJ (2024) A Field Guide to the Stag Beetles of Taiwan. Yuan-Liou Publishing Co., Ltd., Taipei, 191 pp. [in Chinese]

[B8] Chi KH (1970) Air temperature climatology of high mountains in Taiwan. Meteorological Bulletin 16(3): 13–23. [in Chinese with English abstract]

[B9] Coyne JA, Orr HA (2004) Speciation. Sinauer Associates, Inc., MA, USA, 545 pp.

[B10] Darwin C (1871) The Descent of Man and Selection in Relation to Sex. John Murray, London, UK, 424 pp. 10.5962/bhl.title.110063

[B11] De Vivo M, Chou MH, Wu SP, Kuan YH, Chen WY, Wang LJ, Morgan B, Phang GJ, Huang JP (2023) Genomic tools for comparative conservation genetics among three recently diverged stag beetles (Lucanus, Lucanidae). Insect Conservation and Diversity 16(6): 1–17. 10.1111/icad.12678

[B12] DeSalle R, Goldstein P (2019) Review and interpretation of trends in DNA barcoding. Frontiers in Ecology and Evolution 7: 302. 10.3389/fevo.2019.00302

[B13] ESRI (2011) ArcGIS Desktop: Release 10. Environmental Systems Research Institute, Redlands, CA.

[B14] Folmer O, Black M, Hoeh W, Lutz R, Vrijenhoek R (1994) DNA primers for amplification of mitochondrial cytochrome c oxidase subunit I from diverse metazoan invertebrates. Molecular Marine Biology and Biotechnology 3: 294–299. 7881515

[B15] Fujita H (2010) The Lucanid Beetles of the World. Mushi-Sha’s Iconographic Series of Insects 6, Mushi-Sha, Tokyo, Japan, 472 pp. [278 pl.]

[B16] Huang SJ (2016) The Daily of Stag Beetles. Mangrove Publications, a division of Cité Publishing Ltd., Taipei, 286 pp. [in Chinese]

[B17] Huang H, Chen CC (2010) Stag beetles of China I. Formosa Eco. Co., Ltd., Taipei, Taiwan, 288 pp. [66 pl.]

[B18] Huang JP, Wu SP, Chen WY, Pham GJ, Kuan YH (2024) Genomic data revealed inbreeding despite a geographically connected stable effective population size since the Holocene in the protected Formosan Long-Arm Scarab beetle, *Cheirotonus formosanus*. The Journal of Heredity 115(3): 292–301. 10.1093/jhered/esae006 38364316

[B19] Imanishi O (1990) A New Species of the Genus Lucanus Scopoli from Formosa (Coleoptera, Lucanidae). Insect and Nature 25(7): 15–18. [In Japanese with brief English description]

[B20] Kalyaanamoorthy S, Minh BQ, Wong TKF, von Haeseler A, Jermiin LS (2017) ModelFinder: Fast model selection for accurate phylogenetic estimates. Nature Methods 14(6): 587–589. 10.1038/nmeth.4285 PMC5453245 28481363

[B21] Katoh K, Standley DM (2013) MAFFT multiple sequence alignment software version 7: Improvements in performance and usability. Molecular Biology and Evolution 30(4): 772–780. 10.1093/molbev/mst010 PMC3603318 23329690

[B22] Klingenberg CP (1998) Heterochrony and allometry: The analysis of evolutionary change in ontogeny. Biological Reviews of the Cambridge Philosophical Society 73(1): 79–123. 10.1111/j.1469-185X.1997.tb00026.x 9569772

[B23] Kumar S, Stecher G, Li M, Knyaz C, Tamura K (2018) MEGA X: Molecular Evolutionary Genetics Analysis across Computing Platforms. Molecular Biology and Evolution 35(6): 1547–1549. 10.1093/molbev/msy096 PMC5967553 29722887

[B24] Kurosawa Y (1966) Descriptions of Two New Species of the Genus Lucanus SCOPOLI from Formosa (Coleoptera, Lucanidae). Bulletin of the National Museum of Nature and Science, Tokyo 9(3): 339–345.

[B25] Larsson A (2014) AliView: A fast and lightweight alignment viewer and editor for large datasets. Bioinformatics (Oxford, England) 30(22): 3276–3278. 10.1093/bioinformatics/btu531 PMC4221126 25095880

[B26] Mayr E, Ashlock PD (1991) Principles of systematic zoology (2^nd^ edn.). McGraw-Hill, Inc., NY, USA, 328 pp.

[B27] Mizunuma T, Nagai S (1994) The Lucanid Beetles of the World. Part of Mushi Sha’s Iconographic Series of Insects, 1^st^ edn., edited by H. Fijita. Mushi-Sha publishers, Tokyo, Japan, 337 pp.

[B28] Moritz C, Cicero C (2004) DNA barcoding: Promise and pitfalls. PLoS Biology 2(10): e354. 10.1371/journal.pbio.0020354 PMC519004 15486587

[B29] Nguyen LT, Schmidt HA, von Haeseler A, Minh BQ (2015) IQ-TREE: A fast and effective stochastic algorithm for estimating maximum-likelihood phylogenies. Molecular Biology and Evolution 32(1): 268–274. 10.1093/molbev/msu300 PMC4271533 25371430

[B30] Palumbi SR (1996a) Nucleic acids II: The polymerase chain reaction. In: Hillis DM, Moritz C, Mable BK (Eds) Molecular systematics. Sinauer Associates, Sunderland, Massachusetts, USA, 205–274.

[B31] Palumbi SR (1996b) What can molecular genetics contribute to marine biogeography. An urchin’s tale. Journal of Experimental Marine Biology and Ecology 203(1): 75–92. 10.1016/0022-0981(96)02571-3

[B32] Papadopoulou A, Anastasiou I, Vogler AP (2010) Revisiting insect mitochondrial molecular clock: The Mid-Aegean Trench calibration. Molecular Biology and Evolution 27(7): 1659–1672. 10.1093/molbev/msq051 20167609

[B33] Rambaut A, Drummond AJ, Xie D, Baele G, Suchard MA (2018) Posterior Summarization in Bayesian Phylogenetics Using Tracer 1.7. Systematic Biology 67(5): 901–904. 10.1093/sysbio/syy032 PMC6101584 29718447

[B34] Ronquist F, Teslenko M, van der Mark P, Ayres DL, Darling A, Höhna S, Larget B, Liu L, Suchard MA, Huelsenbeck JP (2012) MrBayes 3.2: Efficient Bayesian phylogenetic inference and model choice across a large model space. Systematic Biology 61(3): 539–542. 10.1093/sysbio/sys029 PMC3329765 22357727

[B35] Sakaino H, Yu CK (1993) Some notes on stag-beetles from Taiwan, with descriptions of two new subspecies (Coleoptera, Lucanidae). Gekkan-Mushi 272: 13–16.

[B36] Scopoli JA (1763) Entomologia Carniolica exhibens insecta Carnioliae indigena et distributa in ordines, genera, species, varietates, methodo linnaeana. Ioannis Thomae Trattner, Vienna, Austria, 420 pp. [3 pls.] 10.5962/bhl.title.34434

[B37] Stebbins GL (1959) The role of hybridization in evolution. Proceedings of the American Philosophical Society 103(2): 231–251.

[B38] Tong YJ, Zhang MG, Shaw JJ, Wan X, Yang XK, Bai M (2021) A geometric morphometric dataset of stag beetles. Shengwu Duoyangxing 29(9): 1159–1164. 10.17520/biods.2021160

[B39] Truett GE, Heeger P, Mynatt RL, Truett AA, Walker JA, Warman ML (2000) Preparation of PCR-Quality Mouse Genomic DNA with Hot Sodium Hydroxide and Tris (HotSHOT). BioTechniques 29(1): 52–54. 10.2144/00291bm09 10907076

[B40] Tsai CL, Yeh WB (2016) Subspecific Differentiation Events of Montane Stag Beetles (Coleoptera, Lucanidae) Endemic to Formosa Island. PLoS ONE 11(6): e0156600. 10.1371/journal.pone.015660 PMC4892689 27257861

[B41] Vences M, Thomas M, Van der Meijden A, Chiari Y, Vieites DR (2005) Comparative performance of the 16S rRNA gene in DNA barcoding of amphibians. Frontiers in Zoology 2(1): 1–12. 10.1186/1742-9994-2-5 PMC555853 15771783

[B42] Wang SY (1990) Illustrations of Stag Beetles in Taiwan. Taiwan Museum, Taipei, 189 pp. [in Chinese]

[B43] Wang SY (1994) Illustrations of Stag Beetles in Taiwan: To Learn about Insects of Taiwan V (Coleoptera, Lucanidae). Shu Shin Book Co., Taipei County, 207 pp. [in Chinese]

[B44] Wild AL, Maddison DR (2008) Evaluating nuclear protein-coding genes for phylogenetic utility in beetles. Molecular Phylogenetics and Evolution 48(3): 877–891. 10.1016/j.ympev.2008.05.023 18644735

