# Supplementary material for: Systematic revision and biogeography of the endemic Lucanus kanoi species complex (Coleoptera, Lucanidae) from Taiwan, with the description of a new subspecies
Source: Zookeys. 2026 Jan 22;1267:77–117. doi: 10.3897/zookeys.1267.160494 (PMC12856485; doi:10.3897/zookeys.1267.160494)
Supplement: Supplementary material 1 — Examined sample size and morphological data of this study [file zookeys-1267-077_article-160494__-s001.docx]

**Suppl. material 1.** Examined sample size and morphological data of this study.

| Species | *L. k. kanoi* | *L.* *piceus* | *L. k. kavulunganus* subsp. nov. | *L. ogakii* |
| --- | --- | --- | --- | --- |
| **Male** | 359* | 228* | 38 | 254 |
| “yellowish plaque at femur” | 75 | 30 | 12 | 3 |
| Body length (avg.±SD) | 39.92±4.77 | 38.17±4.48 | 36.22±3.69 | 33.39±3.34 |
| max. | 52.86 | 51.05 | 46.79 | 42.53 |
| min. | 30.30 | 28.15 | 28.07 | 25.04 |
| **Female** | 48 | 33 | 7 | 41 |
| Body length (avg. ±SD) | 32.39±3.93 | 33.82±3.27 | 34.53±2.44 | 30.82±3.48 |
| max. | 43.97 | 39.95 | 37.17 | 38.99 |
| min. | 24.58 | 29.00 | 30.9 | 23.93 |

* Include holotype from NMNS (Tokyo, JP)
