# Supplementary material for: Systematic revision and biogeography of the endemic Lucanus kanoi species complex (Coleoptera, Lucanidae) from Taiwan, with the description of a new subspecies
Source: Zookeys. 2026 Jan 22;1267:77–117. doi: 10.3897/zookeys.1267.160494 (PMC12856485; doi:10.3897/zookeys.1267.160494)
Supplement: Supplementary material 2 — Permission numbers for scientific study of government agencies [file zookeys-1267-077_article-160494__-s002.docx]

**Suppl. material 2**. Permission numbers for scientific study of government agencies.

|  | Permission Numbers | |
| --- | --- | --- |
| Year | County / City Government | Forestry and Nature Conservation Agency |
| 2020 | 1092892956, 1090042207, 1090048062, 1090064338, 10909830000, 1090062928, 10901358600, 1090514074, 1093345690, 1090343005, 1090088363, 1090344663, 1090009540, 1090057227, 1090053380, 1090064592 | 1097151400, 1093162091, 1098161884, 1094162179, 1096162075, 1092104059, 1095162190, 1091152246 |
| 2021 | 1100038766, 1100047058, 1100048680, 1100045193, 1100038471, 1100511347, 1100044170, 1100067302, 11007650400, 11000900400, 1100340014, 1103344547, 1100045660, 1100280205, 1100032126 | 1105161638, 1102102787, 1106161545, 1104161635, 1108161347, 1107151233, 1103161386, 1101151707 |
| 2022 | 1110083565, 1110070156, 1110457727, 1110051100, 1110069484, 1110081770, 11113450400, 1110066201, 1110088530, 11101598800, 1110520512, 1110124892, 1113360218, 1110348147, 1110082232 | 1113107345, 1118107343, 1114104889, 1116108212, 1112113455, 1115110488, 1111110840, 1117340585 |
| 2023 | 1120057587, 1120069555, 1120060109, 1120514381, 1120055350, 1120084943, 11201094400, 11209064300, 1120341824, 1123335917, 1120057942, 1120322640, 1120046456, 1120044982, 1120046206 | 1122112666, 1121110198, 1122114006, 1123111079, 1124103255, 1126106708, 1125110280, 1127103390 |
| 2024 | 1130064537, 1130063643, 1130064860, 1133303862, 1130049176, 1130040128, 1130090574, 1130062686, 1130516352, 11301212200, 11310704300, 1130343603, 1130066047, 1130382108, 1130052638 | 1137112323, 1137390145, 1136109633, 1133111196, 1132112983, 1131110436, 1134103748, 1135112560 |
| 2017 | Taroko National Park | |
|  | 201705070037 | |
| 2021 | Yushan National Park | |
|  | 1100003321 | |
