# Supplementary material for: Systematic revision and biogeography of the endemic Lucanus kanoi species complex (Coleoptera, Lucanidae) from Taiwan, with the description of a new subspecies
Source: Zookeys. 2026 Jan 22;1267:77–117. doi: 10.3897/zookeys.1267.160494 (PMC12856485; doi:10.3897/zookeys.1267.160494)
Supplement: Supplementary material 3 — The acronyms of museums included in this study [file zookeys-1267-077_article-160494__-s003.docx]

**Suppl. material 3**. The acronyms of museums included in this study.

| Museum | Acronyms | Species examined | Sample size (gender) |
| --- | --- | --- | --- |
| National Museum of Nature and Science, Tokyo | NMNS (JP) | *L. k. kanoi* | 1 (M) |
|  |  | *L. piceus* | 1 (M) |
| National Taiwan Museum | NTM | *L. k. kanoi* | 2 (M) |
|  |  | *L. ogakii* | 8 (M) |
| National Museum of Natural Science, Taiwan | NMNS (TW) | *L. k. kanoi* | 14 (10M/4F) |
| Muh Sheng Museum of Entomology | MSME | *L. ogakii chuyunshanus* | 4 (2M/2F) |
| Taiwan Agricultural Research Institute | TARI | *L. k. kanoi* | 10 (9M/1F) |
|  |  | *L. k. kavulunganus* | 10 (9M/1F) |
|  |  | *L. piceus* | 12 (10M/2F) |
|  |  | *L. ogakii* | 12 (10M/2F) |
| Lab. of the first author & Biological Museum of University of Taipei | BMUT | *L. k. kanoi* | 120 (110M/10F) |
|  |  | *L. k. kavulunganus* | 10 (9M/1F) |
|  |  | *L. piceus* | 135 (120M/15F) |
|  |  | *L. ogakii* | 130 (115M/15F) |
