# Supplementary material for: Systematic revision and biogeography of the endemic Lucanus kanoi species complex (Coleoptera, Lucanidae) from Taiwan, with the description of a new subspecies
Source: Zookeys. 2026 Jan 22;1267:77–117. doi: 10.3897/zookeys.1267.160494 (PMC12856485; doi:10.3897/zookeys.1267.160494)
Supplement: Supplementary material 4 — Collectibles of “L. o. chuyunshanus” from MSME [file zookeys-1267-077_article-160494__-s004.docx]

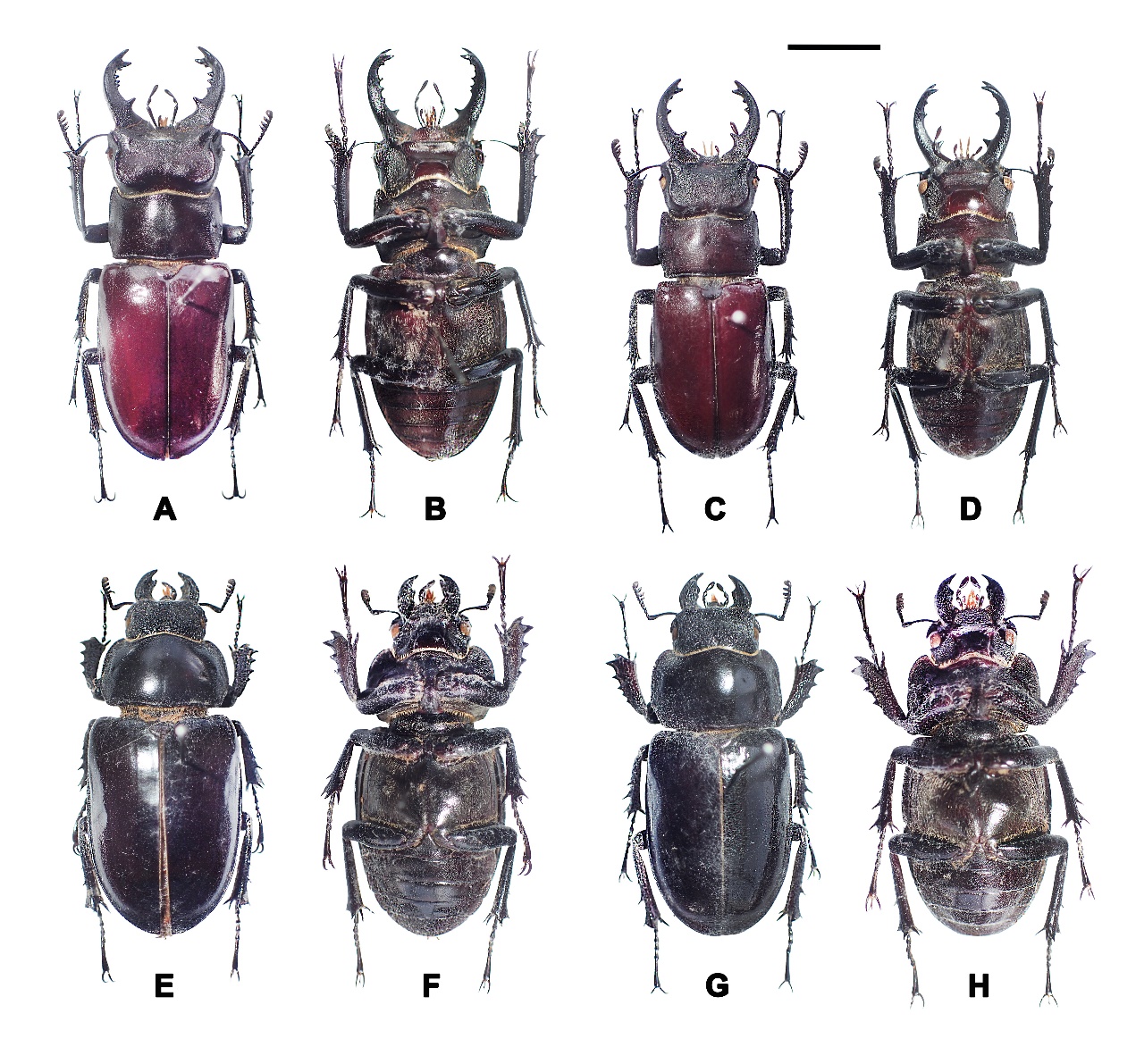


**Suppl. material 4.** Collectibles of “*L. o. chuyunshanus*” from MSME. Scale bar: 1 cm. (The male femur lacks the yellowish plaque, in contrast to the characteristic described for the holotype in the original article.)
