# Supplementary material for: Systematic revision and biogeography of the endemic Lucanus kanoi species complex (Coleoptera, Lucanidae) from Taiwan, with the description of a new subspecies
Source: Zookeys. 2026 Jan 22;1267:77–117. doi: 10.3897/zookeys.1267.160494 (PMC12856485; doi:10.3897/zookeys.1267.160494)
Supplement: Supplementary material 5 — GenBank accession numbers of DNA sequences used in this study [file zookeys-1267-077_article-160494__-s005.docx]

**Suppl. material 5**. GenBank accession numbers of DNA sequences used in this study.

| Species | Sampled locality | Gene sequences | No. of GenBank |
| --- | --- | --- | --- |
| *Lucanus k. kanoi* | Songgang / SG  (Type locality) | mtDNA CO1 | PX279234 |
|  |  | mtDNA 16S | PX279464 |
|  |  | Wingless (Wnt) | PX310144 |
| *L. kanoi kavulunganus* subsp. nov. | Mt. Beidawushan / BDW  (Type locality) | mtDNA CO1 | PX279233 |
|  |  | mtDNA 16S | PX279463 |
|  |  | Wingless (Wnt) | PX310143 |
| *Lucanus k. kanoi* | Jyunda logging-trail / JD | mtDNA CO1 | PX279235 |
|  |  | mtDNA 16S | PX279465 |
|  |  | Wingless (Wnt) | PX310145 |
| *Lucanus k. kanoi* | Mt. Lidongshan / LDS | mtDNA CO1 | PX279236 |
|  |  | mtDNA 16S | PX279466 |
|  |  | Wingless (Wnt) | PX310146 |
| *Lucanus k. kanoi* | Meifeng / MF | mtDNA CO1 | PX279237 |
|  |  | mtDNA 16S | PX279467 |
|  |  | Wingless (Wnt) | PX310147 |
| *Lucanus k. kanoi* | Mt. Lalashan / LLS | mtDNA CO1 | PX279238 |
|  |  | mtDNA 16S | PX279468 |
|  |  | Wingless (Wnt) | PX310148 |
| *L. piceus* | Siji / SJ  (Type locality) | mtDNA CO1 | PX279245 |
|  |  | mtDNA 16S | PX279475 |
|  |  | Wingless (Wnt) | PX310155 |
| *L. piceus* | Sihyuanyakou / SYYK | mtDNA CO1 | PX279243 |
|  |  | mtDNA 16S | PX279473 |
|  |  | Wingless (Wnt) | PX310153 |
| *L. piceus* | Yuanyang Lake / YYL | mtDNA CO1 | PX279246 |
|  |  | mtDNA 16S | PX279476 |
|  |  | Wingless (Wnt) | PX310156 |
| *L. piceus* | Mt. Taipingshan / TPS | mtDNA CO1 | PX279247 |
|  |  | mtDNA 16S | PX279477 |
|  |  | Wingless (Wnt) | PX310157 |
| *L. piceus* | Mt. Tielikushan / TLS | mtDNA CO1 | PX279242 |
|  |  | mtDNA 16S | PX279472 |
|  |  | Wingless (Wnt) | PX310152 |
| *L. piceus* | Mt. Beichatianshan / BCT | mtDNA CO1 | PX279244 |
|  |  | mtDNA 16S | PX279474 |
|  |  | Wingless (Wnt) | PX310154 |
| *L. ogakii* | Bilu Sacred Tree / BL  (Type locality) | mtDNA CO1 | PX279240 |
|  |  | mtDNA 16S | PX279470 |
|  |  | Wingless (Wnt) | PX310150 |
| *L. ogakii* | Ruisui logging-trail / RS | mtDNA CO1 | PX279241 |
|  |  | mtDNA 16S | PX279471 |
|  |  | Wingless (Wnt) | PX310151 |
| *L. ogakii* | Siangyang / SY | mtDNA CO1 | PX279239 |
|  |  | mtDNA 16S | PX279469 |
|  |  | Wingless (Wnt) | PX310149 |
| *L. swinhoei* | Meifeng / MF | mtDNA CO1 | PX279232 |
|  |  | mtDNA 16S | PX279462 |
|  |  | Wingless (Wnt) | PX310142 |
| *L. formosanus* | Mt. Tamanshan / TMS | mtDNA CO1 | PX279231 |
|  |  | mtDNA 16S | PX279461 |
|  |  | Wingless (Wnt) | PX310141 |
| *Neolucanus swinhoei* | Mt. Tamanshan / TMS | mtDNA CO1 | PX279230 |
|  |  | mtDNA 16S | PX279460 |
|  |  | Wingless (Wnt) | PX310140 |
