# Supplementary material for: Systematic revision and biogeography of the endemic Lucanus kanoi species complex (Coleoptera, Lucanidae) from Taiwan, with the description of a new subspecies
Source: Zookeys. 2026 Jan 22;1267:77–117. doi: 10.3897/zookeys.1267.160494 (PMC12856485; doi:10.3897/zookeys.1267.160494)
Supplement: Supplementary material 6 — Bayesian phylogenetic relationships of the Lucanus kanoi species complex [file zookeys-1267-077_article-160494__-s006.docx]

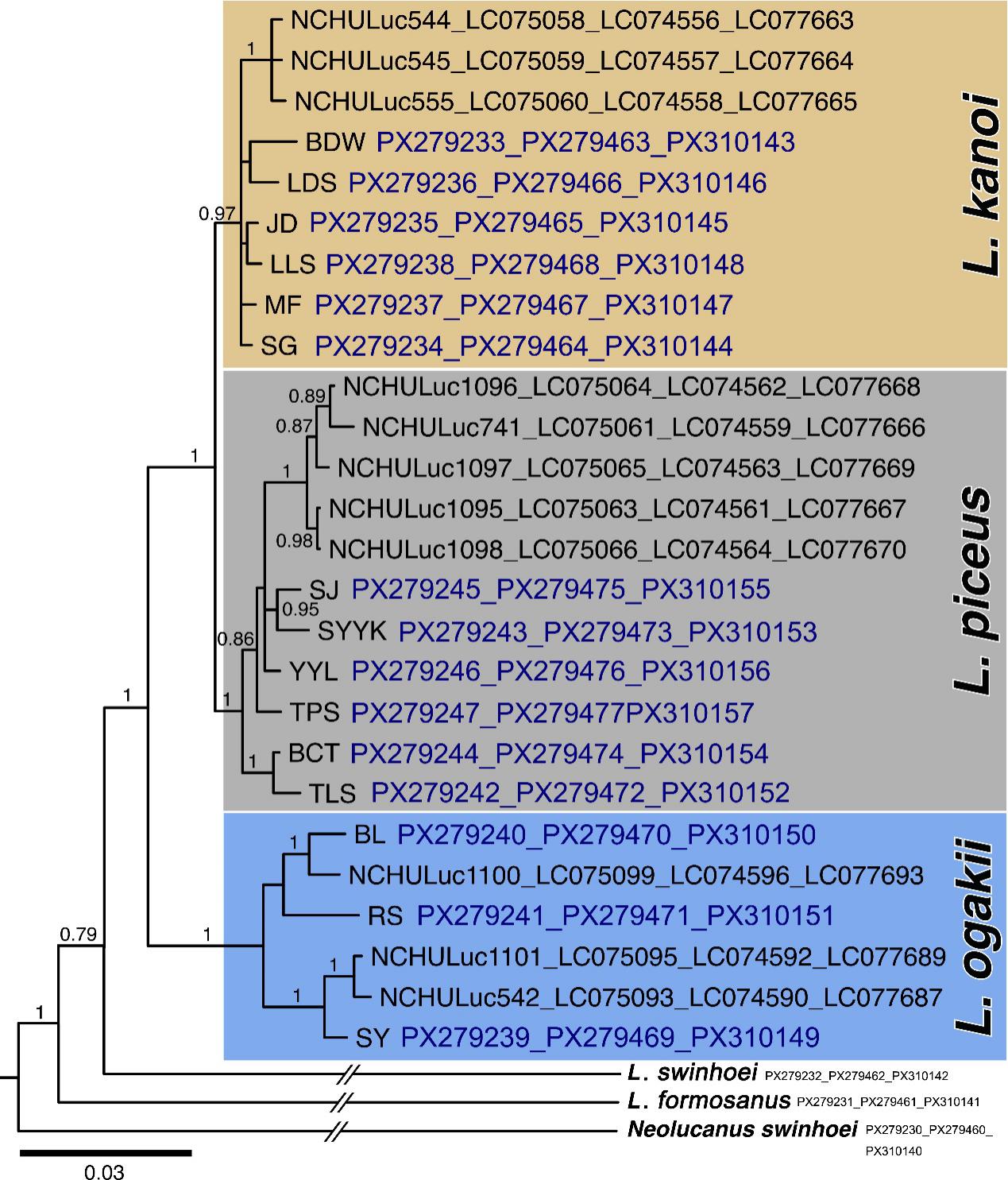


**Suppl. material 6**. Bayesian phylogenetic relationships of the Lucanus kanoi species complex.

Sequences retrieved from GenBank (Tsai & Yeh, 2016) are sequentially labeled with specimen voucher numbers and the corresponding GenBank accession numbers for 16S, COI, and Wnt (black). Numbers above branches indicate Bayesian posterior probabilities greater than 0.7.
