# Supplementary material for: Systematic revision and biogeography of the endemic Lucanus kanoi species complex (Coleoptera, Lucanidae) from Taiwan, with the description of a new subspecies
Source: Zookeys. 2026 Jan 22;1267:77–117. doi: 10.3897/zookeys.1267.160494 (PMC12856485; doi:10.3897/zookeys.1267.160494)
Supplement: Supplementary material 7 — Tree of divergence time estimated with BEAST [file zookeys-1267-077_article-160494__-s007.docx]

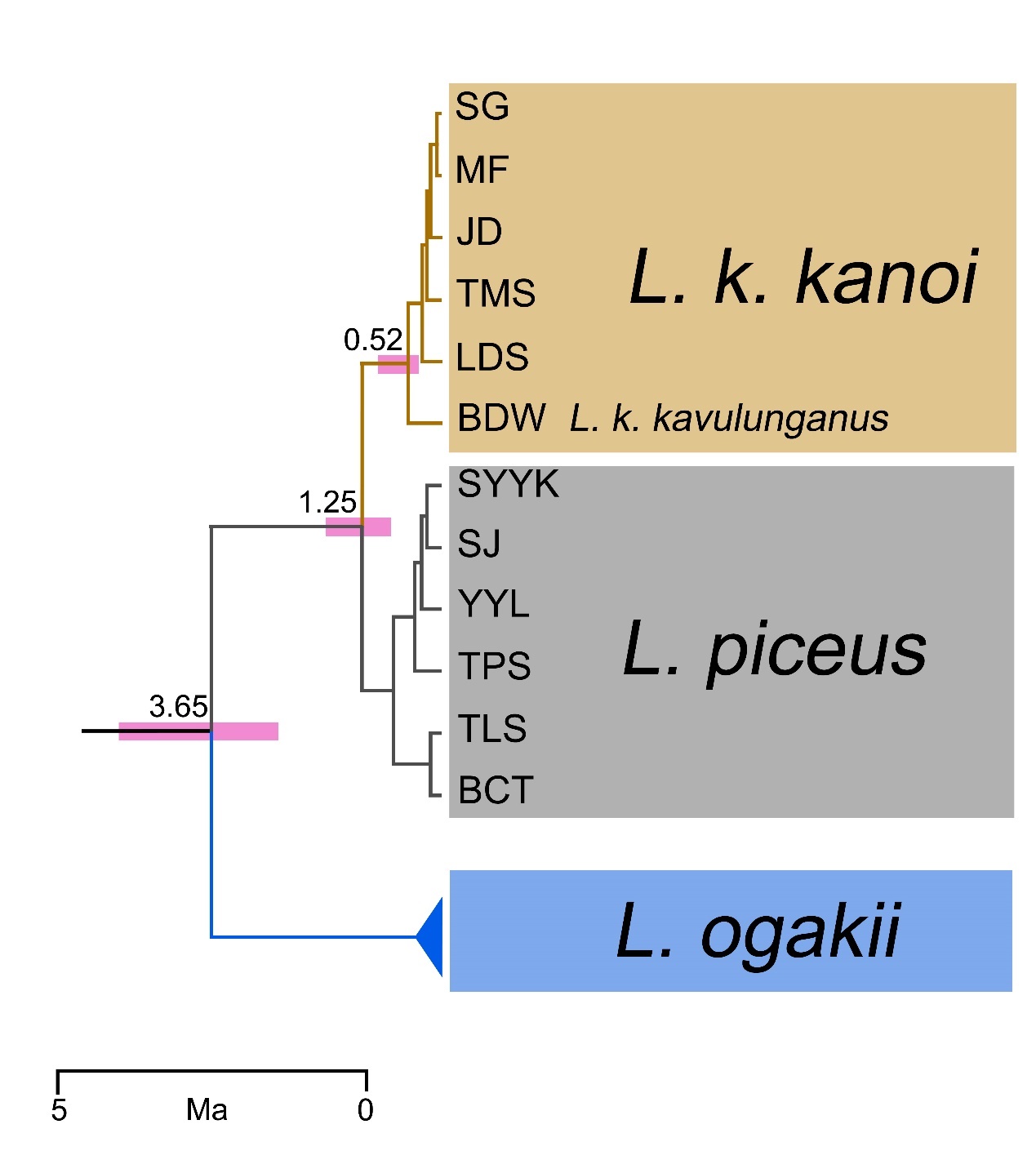


**Suppl. material 7.** Tree of divergence time estimated with BEAST.

The estimated date of the clade that has a Bayesian posterior probability higher than 0.8 is labeled beside the node. Bar, 95% HPD of the date.
