# Supplementary material for: Systematic revision and biogeography of the endemic Lucanus kanoi species complex (Coleoptera, Lucanidae) from Taiwan, with the description of a new subspecies
Source: Zookeys. 2026 Jan 22;1267:77–117. doi: 10.3897/zookeys.1267.160494 (PMC12856485; doi:10.3897/zookeys.1267.160494)
Supplement: Supplementary material 8 — Geographical distribution of the L. kanoi species complex in Taiwan [file zookeys-1267-077_article-160494__-s008.docx]

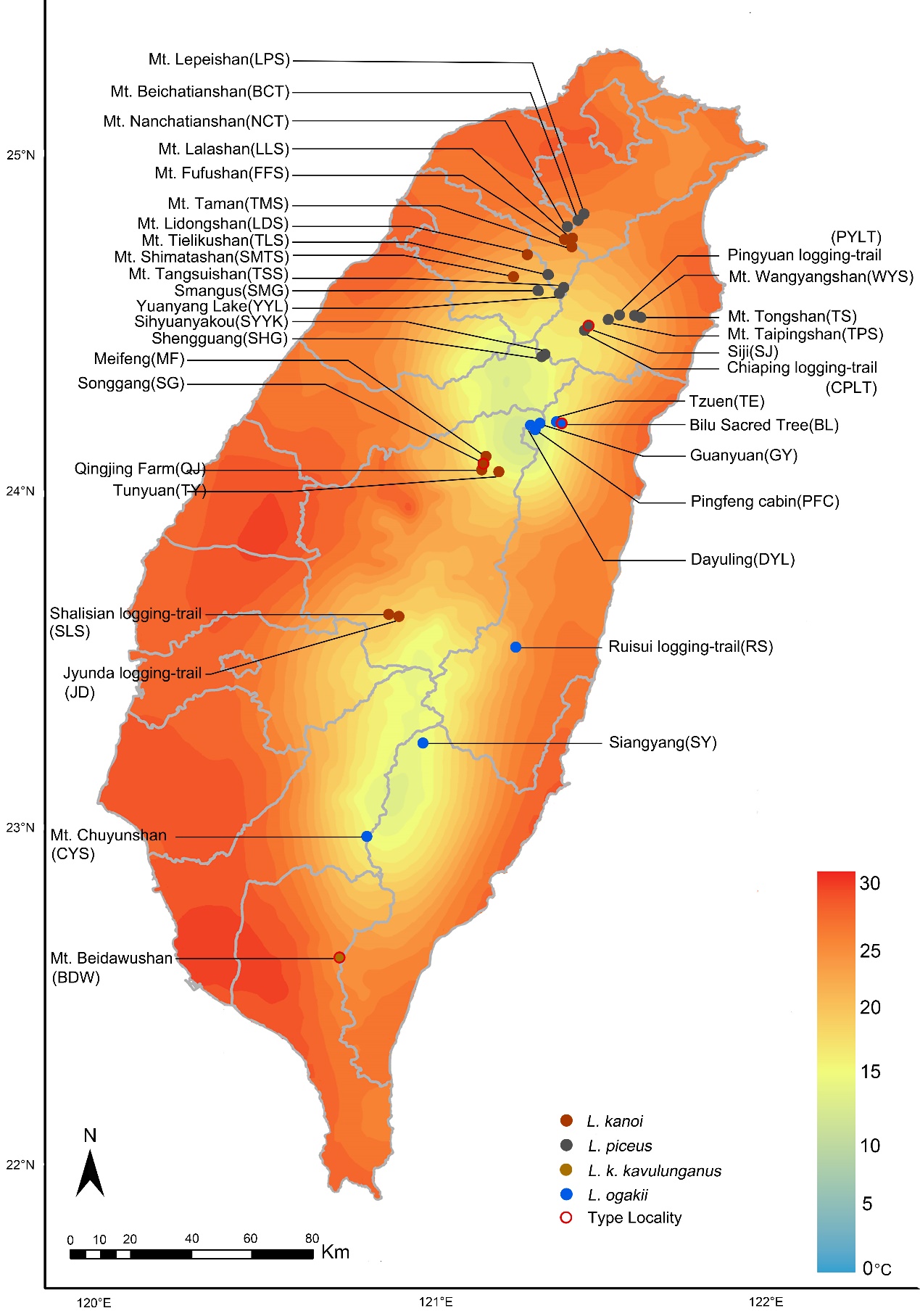


**Suppl. material 8.** Geographical distribution of the L. kanoi species complex in Taiwan, overlaid with mean June temperature isotherms from 2010 to 2020.
