# Supplementary material for: Systematic revision and biogeography of the endemic Lucanus kanoi species complex (Coleoptera, Lucanidae) from Taiwan, with the description of a new subspecies
Source: Zookeys. 2026 Jan 22;1267:77–117. doi: 10.3897/zookeys.1267.160494 (PMC12856485; doi:10.3897/zookeys.1267.160494)
Supplement: Supplementary material 9 — Gap statistic analysis identified two clusters as the optimal solution and three clusters as a suboptimal solution for external morphological data [file zookeys-1267-077_article-160494__-s009.docx]

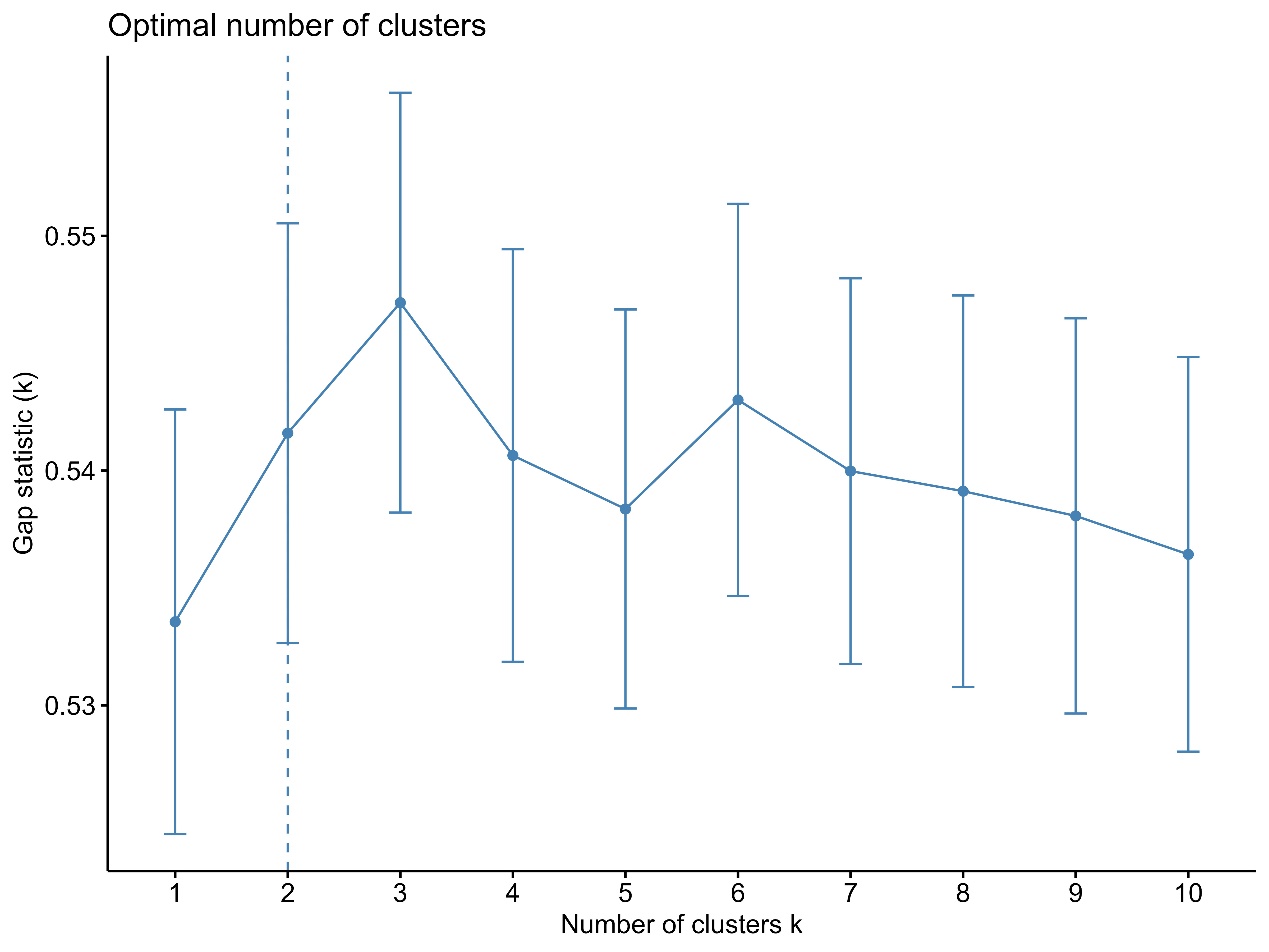


**Suppl. material 9.** Gap statistic analysis identified two clusters as the optimal solution and three clusters as a suboptimal solution for external morphological data.
