# Supplementary material for: Systematic revision and biogeography of the endemic Lucanus kanoi species complex (Coleoptera, Lucanidae) from Taiwan, with the description of a new subspecies
Source: Zookeys. 2026 Jan 22;1267:77–117. doi: 10.3897/zookeys.1267.160494 (PMC12856485; doi:10.3897/zookeys.1267.160494)
Supplement: Supplementary material 10 — Genetic distances among L. kanoi species complex and three outgroup taxa in CO 1 gene (below diagonal) and 16S rRNA gene (above diagonal) [file zookeys-1267-077_article-160494__-s010.docx]

**Suppl. material 10.** Genetic distances among *L. kanoi* species complex and three outgroup taxa in CO 1 gene (below diagonal) and 16S rRNA gene (above diagonal). Numbers on top row refer to species shown on the left column. Genetic distances are shown as percentage.

| Species | 1 | 2 | 3 | 4 | 5 | 6 | 7 | 8 | 9 | 10 | 11 | 12 | 13 | 14 | 15 | 16 | 17 | 18 |
| --- | --- | --- | --- | --- | --- | --- | --- | --- | --- | --- | --- | --- | --- | --- | --- | --- | --- | --- |
| 1 *N. swinhoei* | -- | 0.20313 | 0.29688 | 0.23438 | 0.23438 | 0.21875 | 0.21875 | 0.21875 | 0.21875 | 0.21875 | 0.21875 | 0.21875 | 0.21875 | 0.21875 | 0.21875 | 0.21875 | 0.21875 | 0.21875 |
| 2 *L. formosanus* | 0.15839 | -- | 0.14059 | 0.10277 | 0.07813 | 0.06250 | 0.04688 | 0.09289 | 0.04688 | 0.04688 | 0.04688 | 0.04688 | 0.08515 | 0.08515 | 0.04688 | 0.08498 | 0.08498 | 0.04688 |
| 3 *L. swinhoei* | 0.17702 | 0.15373 | -- | 0.12253 | 0.15625 | 0.14063 | 0.12500 | 0.11067 | 0.12500 | 0.12500 | 0.12500 | 0.12500 | 0.10672 | 0.10672 | 0.12500 | 0.10672 | 0.10672 | 0.12500 |
| 4 *L. ogakii* SY | 0.17702 | 0.16615 | 0.15683 | -- | 0.00000 | 0.01563 | 0.03125 | 0.02959 | 0.03125 | 0.03125 | 0.03125 | 0.03125 | 0.02569 | 0.02569 | 0.03125 | 0.02564 | 0.02564 | 0.03125 |
| 5 *L. ogakii* RS | 0.17857 | 0.16770 | 0.15994 | 0.03571 | -- | 0.01563 | 0.03125 | 0.03125 | 0.03125 | 0.03125 | 0.03125 | 0.03125 | 0.03125 | 0.03125 | 0.03125 | 0.03125 | 0.03125 | 0.03125 |
| 6 *L. ogakii* BL | 0.17547 | 0.16615 | 0.16304 | 0.02950 | 0.02174 | -- | 0.01563 | 0.01563 | 0.01563 | 0.01563 | 0.01563 | 0.01563 | 0.01563 | 0.01563 | 0.01563 | 0.01563 | 0.01563 | 0.01563 |
| 7 *L. piceus* BCT | 0.17702 | 0.15217 | 0.15994 | 0.07143 | 0.07143 | 0.06988 | -- | 0.00000 | 0.00000 | 0.00000 | 0.00000 | 0.00000 | 0.00000 | 0.00000 | 0.00000 | 0.00000 | 0.00000 | 0.00000 |
| 8 *L. piceus* TLS | 0.17702 | 0.15373 | 0.16149 | 0.06677 | 0.06677 | 0.06522 | 0.00466 | -- | 0.00000 | 0.00000 | 0.00000 | 0.00000 | 0.00791 | 0.00791 | 0.00000 | 0.00789 | 0.00789 | 0.00000 |
| 9 *L. piceus* TPS | 0.17857 | 0.15528 | 0.16615 | 0.07143 | 0.07143 | 0.06677 | 0.01553 | 0.01708 | -- | 0.00000 | 0.00000 | 0.00000 | 0.00000 | 0.00000 | 0.00000 | 0.00000 | 0.00000 | 0.00000 |
| 10 *L. piceus* YYL | 0.17702 | 0.15528 | 0.16149 | 0.06522 | 0.07143 | 0.06677 | 0.01242 | 0.01398 | 0.00621 | -- | 0.00000 | 0.00000 | 0.00000 | 0.00000 | 0.00000 | 0.00000 | 0.00000 | 0.00000 |
| 11 *L. piceus* SJ | 0.17547 | 0.15373 | 0.15994 | 0.06366 | 0.07298 | 0.06832 | 0.01708 | 0.01863 | 0.00776 | 0.00466 | -- | 0.00000 | 0.00000 | 0.00000 | 0.00000 | 0.00000 | 0.00000 | 0.00000 |
| 12 *L. piceus* SYYK | 0.17391 | 0.15528 | 0.16304 | 0.06366 | 0.06988 | 0.06832 | 0.02329 | 0.02484 | 0.01398 | 0.01087 | 0.00932 | -- | 0.00000 | 0.00000 | 0.00000 | 0.00000 | 0.00000 | 0.00000 |
| 13 *L. kanoi* LLS | 0.17857 | 0.15217 | 0.16460 | 0.06988 | 0.06988 | 0.06832 | 0.02795 | 0.02950 | 0.02484 | 0.02484 | 0.02329 | 0.02640 | -- | 0.00000 | 0.00000 | 0.00000 | 0.00000 | 0.00000 |
| 14 *L. kanoi* MF | 0.17547 | 0.15528 | 0.16615 | 0.07298 | 0.06988 | 0.07143 | 0.02174 | 0.02640 | 0.02174 | 0.02174 | 0.02329 | 0.02329 | 0.00621 | -- | 0.00000 | 0.00000 | 0.00000 | 0.00000 |
| 15 *L. kanoi* LDS | 0.17857 | 0.15683 | 0.17236 | 0.07609 | 0.06988 | 0.07143 | 0.02795 | 0.02950 | 0.02484 | 0.02795 | 0.02950 | 0.02950 | 0.00932 | 0.00621 | -- | 0.00000 | 0.00000 | 0.00000 |
| 16 *L. kanoi* SG | 0.17702 | 0.15528 | 0.16615 | 0.07298 | 0.06988 | 0.07143 | 0.02329 | 0.02795 | 0.02329 | 0.02329 | 0.02484 | 0.02484 | 0.00776 | 0.00155 | 0.00776 | -- | 0.00000 | 0.00000 |
| 17 *L. kanoi* JD | 0.17857 | 0.15683 | 0.16770 | 0.07298 | 0.06988 | 0.07143 | 0.02484 | 0.02640 | 0.02484 | 0.02484 | 0.02640 | 0.02640 | 0.00621 | 0.00311 | 0.00621 | 0.00466 | -- | 0.00000 |
| 18 *L. kanoi* BDW | 0.18168 | 0.16304 | 0.16925 | 0.07609 | 0.07609 | 0.07764 | 0.03106 | 0.03261 | 0.02795 | 0.02484 | 0.02640 | 0.02640 | 0.01553 | 0.01242 | 0.01242 | 0.01398 | 0.01242 | -- |

Abbreviations: *L*.: *Lucanus*; *N*.: *Neolucanus*; SY: Siangyang; RS: Ruisui; BL: Bilu Sacred Tree; BCT: Beichatianshan; TLS: Tielikushan; TPS: Taipingshan; YYL: Yuanyang Lake; SJ: Siji; SYYK: Sihyuanyakou; LLS: Lalashan; MF: Meifeng; LDS: Lidongshan; SG: Songgang; JD: Jyunda; BDW: Beidawushan.
