# Supplementary material for: Systematic revision and biogeography of the endemic Lucanus kanoi species complex (Coleoptera, Lucanidae) from Taiwan, with the description of a new subspecies
Source: Zookeys. 2026 Jan 22;1267:77–117. doi: 10.3897/zookeys.1267.160494 (PMC12856485; doi:10.3897/zookeys.1267.160494)
Supplement: Supplementary material 12 — Assignment of individuals based on the three-cluster solution from morphological clustering analysis [file zookeys-1267-077_article-160494__-s012.docx]

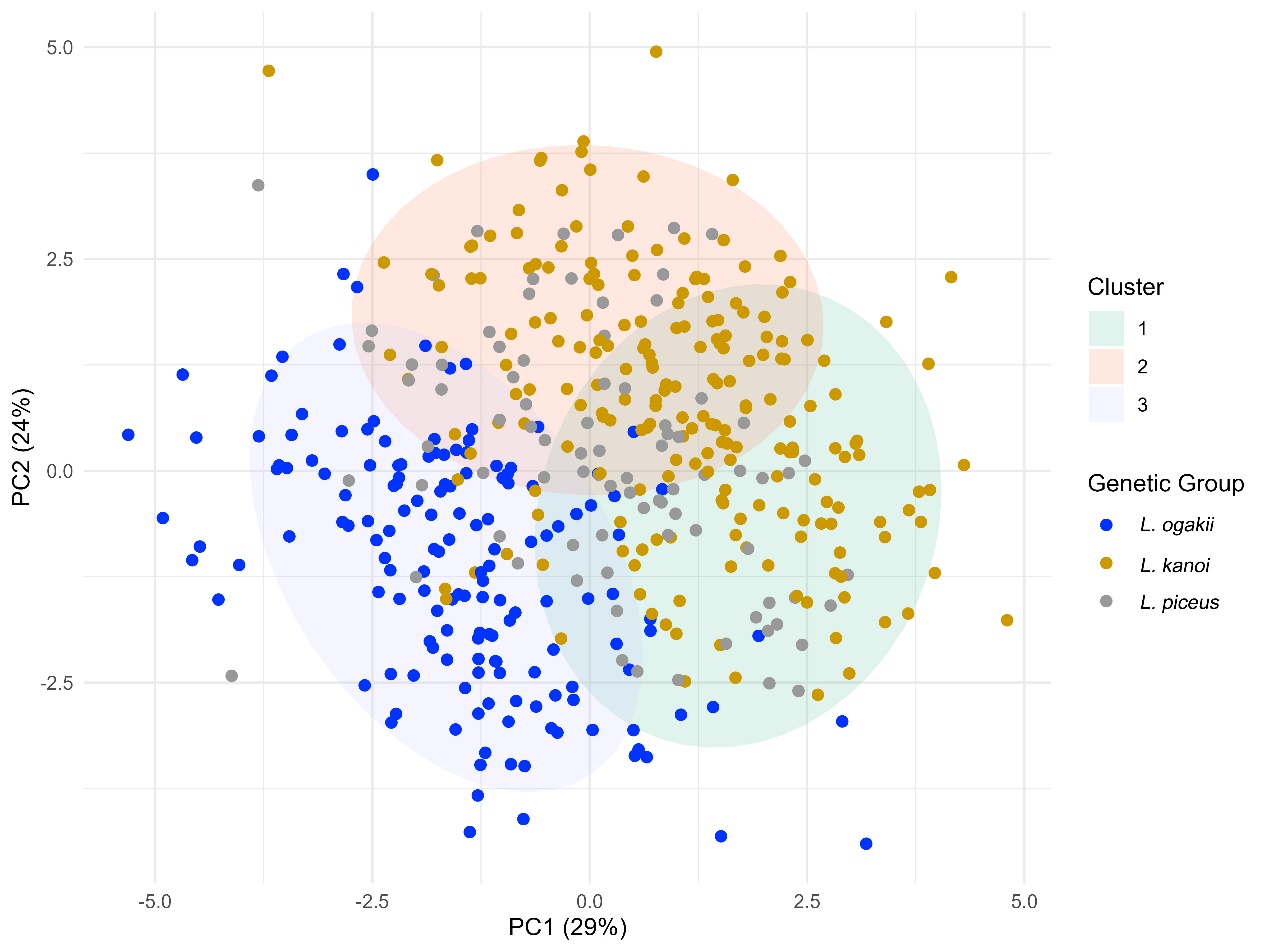


**Suppl. material 12.** Assignment of individuals based on the three-cluster solution from morphological clustering analysis. Cluster 1 included 151 individuals, Cluster 2 had 139, and Cluster 3 comprised 129 individuals. Most L. ogakii specimens (82.6%, n = 128) were assigned to Cluster 1, while the majority of L. kanoi (94.3%, n = 197) and L. piceus (87.1%, n = 74) specimens were distributed across Clusters 2 and 3.
