# Supplementary material for: Systematic revision and biogeography of the endemic Lucanus kanoi species complex (Coleoptera, Lucanidae) from Taiwan, with the description of a new subspecies
Source: Zookeys. 2026 Jan 22;1267:77–117. doi: 10.3897/zookeys.1267.160494 (PMC12856485; doi:10.3897/zookeys.1267.160494)
Supplement: Supplementary material 14 — Genetic distances among L. kanoi species complex and three outgroup taxa in Wnt gene [file zookeys-1267-077_article-160494__-s014.docx]

**Suppl. material 14.** Genetic distances among *L. kanoi* species complex and three outgroup taxa in Wnt gene. Numbers on top row refer to species shown on the left column. Genetic distances are shown as percentage.

| Species | 1 | 2 | 3 | 4 | 5 | 6 | 7 | 8 | 9 | 10 | 11 | 12 | 13 | 14 | 15 | 16 | 17 | 18 |
| --- | --- | --- | --- | --- | --- | --- | --- | --- | --- | --- | --- | --- | --- | --- | --- | --- | --- | --- |
| 1 *N. swinhoei* | -- |  |  |  |  |  |  |  |  |  |  |  |  |  |  |  |  |  |
| 2 *L. formosanus* | 0.14286 | -- |  |  |  |  |  |  |  |  |  |  |  |  |  |  |  |  |
| 3 *L. swinhoei* | 0.14739 | 0.02041 | -- |  |  |  |  |  |  |  |  |  |  |  |  |  |  |  |
| 4 *L. ogakii* SY | 0.14512 | 0.01814 | 0.02494 | -- |  |  |  |  |  |  |  |  |  |  |  |  |  |  |
| 5 *L. ogakii* RS | 0.14512 | 0.02268 | 0.02721 | 0.01587 | -- |  |  |  |  |  |  |  |  |  |  |  |  |  |
| 6 *L. ogakii* BL | 0.15420 | 0.02948 | 0.03628 | 0.01587 | 0.02268 | -- |  |  |  |  |  |  |  |  |  |  |  |  |
| 7 *L. piceus* BCT | 0.14286 | 0.01587 | 0.02268 | 0.00680 | 0.00907 | 0.01361 | -- |  |  |  |  |  |  |  |  |  |  |  |
| 8 *L. piceus* TLS | 0.14739 | 0.02268 | 0.02948 | 0.00907 | 0.01587 | 0.01134 | 0.00680 | -- |  |  |  |  |  |  |  |  |  |  |
| 9 *L. piceus* TPS | 0.14286 | 0.01587 | 0.02494 | 0.00907 | 0.01134 | 0.02041 | 0.00680 | 0.01361 | -- |  |  |  |  |  |  |  |  |  |
| 10 *L. piceus* YYL | 0.14286 | 0.01814 | 0.02041 | 0.00907 | 0.00680 | 0.02041 | 0.00680 | 0.01361 | 0.00907 | -- |  |  |  |  |  |  |  |  |
| 11 *L. piceus* SJ | 0.14739 | 0.02268 | 0.02948 | 0.00907 | 0.01587 | 0.00680 | 0.00680 | 0.00454 | 0.01361 | 0.01361 | -- |  |  |  |  |  |  |  |
| 12 *L. piceus* SYYK | 0.14059 | 0.01814 | 0.02041 | 0.00907 | 0.01134 | 0.01587 | 0.00680 | 0.00907 | 0.00907 | 0.00454 | 0.00907 | -- |  |  |  |  |  |  |
| 13 *L. kanoi* LLS | 0.14286 | 0.01587 | 0.02268 | 0.00680 | 0.00907 | 0.01361 | 0.00000 | 0.00680 | 0.00680 | 0.00680 | 0.00680 | 0.00680 | -- |  |  |  |  |  |
| 14 *L. kanoi* MF | 0.14512 | 0.02268 | 0.02494 | 0.01361 | 0.01134 | 0.01587 | 0.00680 | 0.00907 | 0.01361 | 0.00907 | 0.00907 | 0.00454 | 0.00680 | -- |  |  |  |  |
| 15 *L. kanoi* LDS | 0.14512 | 0.02041 | 0.02721 | 0.00680 | 0.01814 | 0.00907 | 0.00907 | 0.00680 | 0.01134 | 0.01134 | 0.00227 | 0.00680 | 0.00907 | 0.01134 | -- |  |  |  |
| 16 *L. kanoi* SG | 0.14059 | 0.01587 | 0.02268 | 0.00680 | 0.01361 | 0.01814 | 0.00454 | 0.01134 | 0.00680 | 0.00680 | 0.01134 | 0.00680 | 0.00454 | 0.01134 | 0.00907 | -- |  |  |
| 17 *L. kanoi* JD | 0.14286 | 0.01814 | 0.02494 | 0.00907 | 0.00680 | 0.01587 | 0.00227 | 0.00907 | 0.00907 | 0.00907 | 0.00907 | 0.00907 | 0.00227 | 0.00907 | 0.01134 | 0.00680 | -- |  |
| 18 *L. kanoi* BDW | 0.14286 | 0.02041 | 0.02721 | 0.01134 | 0.01814 | 0.02268 | 0.00907 | 0.01134 | 0.01134 | 0.01134 | 0.01587 | 0.01134 | 0.00907 | 0.01587 | 0.01361 | 0.00907 | 0.01134 | -- |

Abbreviations: *L*.: *Lucanus*; *N*.: *Neolucanus*; SY: Siangyang; RS: Ruisui; BL: Bilu Sacred Tree; BCT: Beichatianshan; TLS: Tielikushan; TPS: Taipingshan; YYL: Yuanyang Lake; SJ: Siji; SYYK: Sihyuanyakou; LLS: Lalashan; MF: Meifeng; LDS: Lidongshan; SG: Songgang; JD: Jyunda; BDW: Beidawushan.
